# Gut microbiota and constipation: from causal evidence to therapeutic strategies—a state-of-the-art narrative review

**DOI:** 10.3389/fmicb.2026.1817279

**Published:** 2026-07-20

**Authors:** Yiyuan Chen, Wenqian Duan, Mengdie Du, Miaomiao Guo, Yongshun Sun

**Affiliations:** 1 Shanghai Municipal Hospital of Traditional Chinese Medicine, Shanghai University of Traditional Chinese Medicine, Shanghai, China; 2 Shanghai University of Traditional Chinese Medicine, Shanghai, China

**Keywords:** 5-hydroxytryptamine, brain-gut axis, constipation, gut microbiota, prebiotics, probiotics, short-chain fatty acids

## Abstract

Constipation is a common functional disorder of the gastrointestinal tract with a global prevalence of approximately 10–20%, which seriously affects patients’ quality of life and imposes a heavy socioeconomic burden. In recent years, the role of the gut microbiota in the pathogenesis of constipation has received increasing attention, particularly in the context of the brain-gut axis theory. In this narrative review, we critically examine the research literature on constipation and intestinal microecology published over the past decade, focusing on four aspects: (1) the characteristics of the gut microbiota in patients with constipation, including changes in microbial diversity, alterations in the abundance of specific taxa, and differences across constipation subtypes; (2) Mendelian randomization studies that provide genetic-level evidence consistent with the hypothesis that certain microbiota alterations may precede constipation rather than merely result from it; (3) mechanisms of microbiota-host interactions mediated by the brain-gut axis, with an emphasis on neural, metabolic and immune pathways; and (4) microbiota-based intervention strategies (probiotics, prebiotics, synbiotics, postbiotics and fecal microbiota transplantation) and their clinical evidence. Our findings suggest that specific microbiota alterations may contribute to constipation pathophysiology and the promise of personalized, microbiome-based therapies. Although microbiota-based interventions show potential therapeutic value in selected patients, current evidence is limited by substantial heterogeneity in study design, small sample sizes, inconsistent microbiome signatures, and limited long-term safety data. High-quality evidence from large, well-designed RCTs is lacking for most interventions, and findings from low-certainty studies (e.g., conference abstracts, animal experiments, small uncontrolled trials) should be interpreted as preliminary and hypothesis-generating rather than conclusive. Therefore, microbiota-targeted therapies should currently be considered exploratory or adjunctive rather than established standard treatments for constipation. Future progress will require standardized methodologies, mechanistic validation studies, and phenotype-stratified clinical trials to support translation toward precision microbiome-based medicine.

## 1 Introduction

Constipation is a common functional gastrointestinal disorder characterized by reduced stool frequency (fewer than three bowel movements per week), straining, hard or lumpy stools, and a sensation of incomplete evacuation (Mearin et al., 2016; Bharucha and Lacy, 2020). According to the Rome IV criteria, the diagnosis of functional constipation (FC) requires the presence of at least two of these symptoms during the preceding 3 months, with symptom onset at least 6 months before diagnosis (Drossman and Hasler, 2016). Epidemiological surveys indicate a global prevalence of approximately 10–20%, which increases with age—exceeding 30% in the elderly—and is higher in women (Sperber et al., 2021; Barberio et al., 2021; Yurtdaş et al., 2020). Constipation not only impairs quality of life but also imposes a substantial socioeconomic burden; annual medical expenditures related to constipation in the United States amount to hundreds of millions of dollars, not including over-the-counter laxatives (Harris and Chang, 2022; Schmier et al., 2014).

The etiology of constipation is complex and can be divided into primary (functional) and secondary forms. The pathogenesis of primary constipation involves intestinal dysmotility, visceral hypersensitivity, pelvic floor muscle dysfunction, and abnormalities of the enteric nervous system (ENS) (Bharucha and Wald, 2019; Vriesman et al., 2020). Secondary constipation may be caused by medications (e.g., opioids, antidepressants, calcium channel blockers), endocrine or metabolic diseases (e.g., diabetes mellitus, hypothyroidism), neurological disorders (e.g., Parkinson‘s disease, multiple sclerosis), or organic intestinal diseases (Xu et al., 2021; Yuan et al., 2024).

With the rapid development of high-throughput sequencing and metabolomics, the role of the gut microbiota in constipation has gained increasing attention (Shin et al., 2019; Ohkusa et al., 2019). The human gut harbors approximately 1,000–1,500 microbial species, and the collective microbial genome is more than 150 times larger than the human genome, earning it the description “second genome” or “neglected organ” (Tierney et al., 2019; Clarke et al., 2014). These microorganisms form a symbiotic relationship with the host and maintain intestinal homeostasis by fermenting dietary fiber to produce short-chain fatty acids (SCFAs), participating in bile acid metabolism, and synthesizing neuroactive substances (Rowland et al., 2018; Oliphant and Allen-Vercoe, 2019). Numerous studies have reported gut microbiota alterations in patients with constipation, mainly a decrease in butyrate-producing bacteria, an increase in potentially pathogenic bacteria, and enrichment of methanogens, although findings remain partially inconsistent across populations and study designs (Zhang et al., 2021; Yang et al., 2022).

The brain-gut axis (BGA) provides an important theoretical framework for understanding microbiota-host interactions (Mayer et al., 2022). The BGA is a bidirectional communication network between the central nervous system and the gastrointestinal tract, involving neural, endocrine and immune pathways (Carabotti et al., 2015; Dinan and Cryan, 2017). The gut microbiota, as a key regulator of this axis, can influence gut motility, visceral sensation and barrier function through its metabolites, and can also send signals to the central nervous system via the vagus nerve and other routes, affecting mood and behavior (Cryan et al., 2019; Margolis et al., 2021). The concept of the brain-gut-microbiota axis (BGMA) has elevated the gut microbiota from a passive participant to an active regulator, offering new perspectives on the pathogenesis of functional gastrointestinal disorders such as constipation (Martin et al., 2018; Wan et al., 2025).

Based on this framework, intervention strategies targeting the intestinal microbiota—probiotics, prebiotics, synbiotics, postbiotics and fecal microbiota transplantation (FMT)—have become active research areas for constipation treatment (Dimidi et al., 2020; Quigley, 2019). These strategies aim to regulate microbial composition, enhance metabolite production and improve intestinal barrier function, potentially overcoming the limitations of traditional laxatives that only relieve symptoms without addressing underlying causes (Araújo and Botelho, 2022; Pan et al., 2022).

### 1.1 Literature search strategy

Literature searches were primarily conducted using PubMed, Web of Science, Embase, and supplemented by Google Scholar databases. Searches were performed up to February 2026. The following search term combinations were used: (“constipation” OR “functional constipation” OR “slow-transit constipation”) AND (“gut microbiota” OR “microbiome” OR “dysbiosis”) for microbiota characterization; for MR studies, (“Mendelian randomization” AND “constipation” AND “gut microbiota”); for mechanisms, (“brain-gut axis” OR “short-chain fatty acids” OR “bile acids” OR “serotonin” OR “ICC” OR “macrophage”) AND (“constipation”); for interventions, (“probiotics” OR “prebiotics” OR “synbiotics” OR “postbiotics” OR “fecal microbiota transplantation”) AND (“constipation”). Priority was given to peer-reviewed human studies, meta-analyses, randomized controlled trials (RCTs), mechanistic studies, and recent high-impact reviews published in English.

Inclusion criteria were: (1) human studies, systematic reviews, meta-analyses, or RCTs; (2) studies reporting microbiota composition, mechanistic pathways, or clinical outcomes of microbiota-based interventions in patients with FC; (3) articles published in English with available full text. Exclusion criteria were: (1) studies in non-functional constipation (e.g., secondary constipation due to organic diseases); (2) studies not focusing on gut microbiota or interventions; (3) case reports, editorials, or commentaries. The literature screening process was performed independently by two authors (YC and DW), with disagreements resolved by discussion with a third author (YS).

Given the rapidly evolving nature of microbiome research, selected conference abstracts and preliminary reports from 2025-2026 were also considered for inclusion. The following criteria were applied for grey literature: (1) only abstracts from major gastroenterology and microbiome conferences (including IDDF, UEG Week, DDW, and Gut Microbiota for Health World Summit) were considered; (2) abstracts from small, non-specialized, or regional conferences were excluded; (3) all included conference abstracts were explicitly labeled as preliminary and were not used as primary evidence to support main conclusions, but rather to illustrate emerging trends and generate hypotheses; (4) no formal quality grading was applied to conference abstracts, as they have not undergone full peer review; instead, they are presented with explicit cautionary statements and are clearly distinguished from peer-reviewed evidence in all tables and discussions. Conference abstracts published before 2025 were not included, as they were superseded by peer-reviewed publications or had not been further validated.

### 1.2 Rationale and scope of this review

Although several previous reviews have described the association between gut microbiota and constipation, most have been largely descriptive, with limited attention to causal inference from MR studies, systematic risk-of-bias assessment, or stratified evaluation of clinical evidence. Furthermore, few existing reviews have proposed an integrated conceptual framework linking microbiota alterations, BGA mechanisms, and intervention strategies in a self-reinforcing cycle. To address these gaps, this review provides a critical appraisal of current evidence, including: (1) an evidence-stratified summary of microbiota characteristics across constipation subtypes; (2) a comprehensive assessment of MR studies for genetic-level causal inference; (3) a detailed mechanistic analysis of neural, metabolic and immune pathways; (4) GRADE-based evaluation and risk-of-bias assessment of microbiota-based interventions; and (5) a novel conceptual framework—the microbiota-brain-gut dysregulation loop—to guide future research.

Although traditional cross-sectional studies cannot determine whether microbiota alterations are a cause or a consequence of constipation, emerging Mendelian randomization (MR) studies suggest that specific microbial changes may precede constipation and could contribute to its pathogenesis. This review critically appraises this new evidence, discusses the major sources of heterogeneity in the field, and proposes a microbiota-driven mechanistic framework for constipation.

Therefore, the unique contribution of this review is not merely to summarize emerging evidence, but to critically appraise evidence certainty, integrate causal inference and mechanistic pathways, and propose a conceptual framework to guide future research.

### 1.3 Organization of this review

This review is organized as follows: Section 2 characterizes gut microbiota alterations in patients with constipation, including taxonomic changes, subtype differences (Section 2.4), and causal evidence from MR studies (Section 2.5); Section 3 discusses the BGA mechanisms through which the microbiota influences intestinal motility; Section 4 evaluates microbiota-based intervention strategies (probiotics, prebiotics, synbiotics, postbiotics and FMT) with evidence stratification; Section 5 provides a critical appraisal of current evidence, proposes a novel mechanistic framework (the microbiota-brain-gut dysregulation loop), and outlines future research directions and potential clinical implications.

## 2 Characteristics of the gut microbiota in patients with constipation

### 2.1 Microbial diversity and compositional changes

The composition of the gut microbiota differs significantly between patients with constipation and healthy individuals. Several studies have analyzed fecal microbiota using 16S rRNA gene sequencing; although findings remain partially heterogeneous, several recurring patterns have been reported.

Alpha diversity reflects within-sample richness and evenness. Some studies reported reduced alpha diversity in constipated patients. Mancabelli et al. (2017) (147 subjects: 68 constipated, 79 controls) found that the Shannon index was significantly lower in constipated patients. Zhuang et al. (2019) observed similar results in 20 patients with FC and 20 controls. However, other studies did not find significant differences. Han et al. (2024), using metagenomic analysis of 24 slow-transit constipation (STC) patients and 24 controls, reported no significant differences in Shannon or Simpson indices. This inconsistency may be attributable to differences in constipation subtype, disease duration, age, and DNA extraction methods (Lim et al., 2018) (see Section 5.1 for detailed discussion).

Beta diversity reflects structural differences between samples. Most studies consistently show significant separation between constipated patients and healthy controls. Fan et al. (2022) (30 STC patients, 30 controls) found that principal coordinate analysis (PCoA) clearly separated the two groups, with PERMANOVA confirming significant differences (*R*^2^ = 0.134, *p* = 0.001). Similar results were obtained by Han et al. (2024) using non-metric multidimensional scaling (NMDS; *R*^2^ = 0.050, *p* < 0.001). Thus, constipation appears to be associated with an altered microbial composition, although specific signatures remain heterogeneous.

### 2.2 Compositional changes at phylum/genus level

The most consistent compositional finding across multiple studies is a reduction in butyrate-producing bacteria. Several observational studies and meta-analyses have reported decreased abundances of key butyrate producers such as *Faecalibacterium*, *Roseburia*, *Coprococcus*, and the *Eubacterium rectale* group in patients with constipation (Zhuang et al., 2019; Han et al., 2024; Louis and Flint, 2017; Zhou et al., 2024; Tian et al., 2021). Supportive evidence for a potential causal direction comes from a two-sample bidirectional MR study by Zhou et al. (2024), which found that *Coprococcus* abundance was negatively associated with constipation risk (OR = 0.74, 95% CI 0.64–0.86, *p* = 0.0001); reverse MR analysis suggested that constipation itself did not significantly alter microbial composition, which is consistent with, but does not prove, a directional effect. Additional observational studies have reinforced this pattern: Han et al. (2024) reported reduced *Coprococcus comes* and *Roseburia intestinalis* in STC patients using metagenomics, and Tian et al. (2021) similarly observed decreased *R. intestinalis*. In FC, Zhuang et al. (2019) found significantly reduced *Faecalibacterium* and *Roseburia*.

Increased potentially pathogenic bacteria is another feature. Zhou et al. (2024) found that abundance of the phylum Bacteroidetes was positively associated with constipation risk (OR = 1.22, 95% CI 1.00–1.50, *p* = 0.04). Fan et al. (2022) reported that *Bacteroides*, *Parabacteroides*, *Desulfovibrionaceae* and *Ruminiclostridium* were significantly up-regulated, while *Subdoligranulum* was down-regulated in STC. Roy et al. (2025) noted that in constipation-predominant irritable bowel syndrome (IBS-C), Enterobacteriaceae and *Escherichia coli* were increased.

The relationship between methanogens and constipation has been investigated extensively, but findings remain contradictory and context-dependent. *Methanobrevibacter smithii* is the predominant methanogenic archaeon in the human gut, and experimental evidence suggests that methane inhibits intestinal peristalsis (Hoegenauer et al., 2022). Low-certainty evidence from a small RCT (Culqui Lévano et al., 2025, *n* = 24) reported that a reduction in *M. smithii* after synbiotic supplementation was accompanied by improved stool consistency (Culqui Lévano et al., 2025). In contrast, moderate-certainty genetic evidence from a MR study by Zheng J. et al. (2025) found a negative correlation between methanobacteria abundance and constipation risk (higher abundance associated with lower risk). This apparent contradiction suggests that the relationship between *M. smithii* and constipation is complex and may depend on host genetics, diet, or other microbial interactions (see Section 5.1 for critical analysis).

Changes in *Bifidobacterium* and *Lactobacillus* are controversial. Some studies reported decreases in constipated patients (He et al., 2022; Zhu et al., 2014), while others found no significant differences. This inconsistency may be related to DNA extraction methods: methods that include a bead-beating (wall-breaking) step extract DNA more efficiently from Gram-positive bacteria, resulting in higher detected abundances of Firmicutes and *Bifidobacterium* (Lim et al., 2018). Erhardt et al. (2023) suggested that differences in DNA extraction methods are an important source of heterogeneity.

### 2.3 Microbiota characteristics of different constipation subtypes

FC is not a single entity; according to Rome IV criteria it can be classified as normal-transit, slow-transit, or defecatory disorder subtypes, in addition to IBS-C (Schmulson and Drossman, 2017). Evidence suggests that microbiota profiles differ across constipation subtypes, although few studies have directly compared subtypes within the same cohort.

STC is characterized by delayed colonic transit. A metagenomic analysis by Han et al. (2024) showed that *Gordonibacter pamelaeae*, *Bifidobacterium longum*, Firmicutes bacterium CAG 94 and *Anaerotruncus colihominis* were increased in STC, whereas *C. comes* and *R. intestinalis* were decreased. A multi-omics analysis by Fan et al. (2022) revealed up-regulation of *Bacteroides*, *Parabacteroides*, *Desulfovibrionaceae* and *Ruminiclostridium*, down-regulation of *Subdoligranulum*, and significant alterations in bile acid metabolism pathways.

IBS-C may show a different profile. Roy et al. (2025) noted that *E. coli* and Enterobacteriaceae were increased, while *Bifidobacterium* and *Lactobacillus* were decreased in IBS-C. Chassard et al. (2012) found a decrease in the butyrate-producing *Roseburia-Eubacterium rectale* group and an increase in lactate- and hydrogen-utilizing sulfate-reducing bacteria in IBS-C.

Mucosa-associated versus fecal microbiota differ. Parthasarathy et al. (2016) compared colonic mucosal and fecal microbiota in constipated patients and healthy controls, finding significant differences: mucosal microbiota was more closely associated with constipation symptoms, whereas fecal microbiota correlated better with colonic transit time and methane production. Quigley and Spiller (2016) noted that fecal microbiota may not fully reflect mucosal characteristics due to the drier stools of constipated patients.

Beyond STC and IBS-C, normal-transit constipation (NTC) and defecatory disorder (DD) represent additional subtypes under the Rome IV classification. In the following subsection, we synthesize recent evidence comparing microbial signatures across all four subtypes, including direct head-to-head comparisons that were not available in earlier studies.

### 2.4 Comparative microbiota features across constipation subtypes

Beyond STC and IBS-C, normal-transit constipation (NTC) and defecatory disorder (DD) represent additional subtypes under the Rome IV classification, each with potentially distinct microbial signatures. A prospective cohort study by Yu et al. (2023) compared fecal microbiota between 31 STC patients and 22 NTC patients, finding that STC patients had lower relative abundance of *Bacteroidaceae* and higher abundance of *Peptostreptococcaceae*, Christensenellaceae and Clostridiaceae compared with NTC patients (Yu et al., 2023). Moreover, in 28 patients with DD, the relative abundance of *Bacteroidaceae* and Ruminococcaceae was higher than in non-DD patients (Yu et al., 2023). A more recent study by Hu et al. (2025) using 16S sequencing and metabolomics in 60 FC patients (20 with delayed colonic transit time, 20 with normal colonic transit time, and 20 healthy controls) identified key bacteria distinguishing different colonic transit patterns: *Alistipes*, *Akkermansia*, *Oscillibacter* and *Ruthenibacterium* were enriched in patients with delayed colonic transit time, whereas *Roseburia* was a key bacterium in patients with normal colonic transit time (Hu et al., 2025). Notably, *Roseburia* showed a positive correlation with butyrate and acetate levels (FDR < 0.05), suggesting that microbial differences across transit subtypes may translate into functional differences in SCFA production (Hu et al., 2025).

A comprehensive stratified study by Zhu et al. (2025) further characterized microbial profiles across age groups and constipation subtypes. The study found that microbial richness and diversity were higher in STC than in the DD group, with STC enriched in taxa associated with slower peristalsis (e.g., taxa increasing sphincter tone and inhibiting intestinal peristalsis) and DD showing enrichment of motility-promoting taxa (Zhu et al., 2025). These findings underscore the pathophysiological heterogeneity of FC and support the development of subtype-specific microbiota-targeted therapies.

Collectively, these studies indicate that different constipation subtypes—STC, NTC, DD and IBS-C—exhibit distinct gut microbial signatures. However, direct head-to-head comparisons remain limited, and most studies have been small, single-center, with heterogeneous methodologies. Future large-scale, multi-center studies with uniform subtype classification are needed to validate these subtype-specific microbial signatures and to explore whether they can guide personalized treatment selection.

### 2.5 From association to causation: insights from MR

Having described the compositional alterations in gut microbiota across different constipation subtypes, a fundamental question arises: are these microbiota changes a cause of constipation, or merely a consequence of altered intestinal transit and stool retention? Traditional cross-sectional studies cannot answer this question. MR uses genetic variants as instrumental variables, which can overcome confounding and reverse causality to some extent, providing stronger evidence for causal inference (Smith and Ebrahim, 2003). It is important to emphasize, however, that MR provides genetic-level inference rather than proof of biological causation; the findings discussed below should be interpreted as hypothesis-generating and require experimental validation.

The two-sample bidirectional MR study by Zhou et al. (2024) analyzed the causal relationship between gut microbiota and constipation. Using inverse-variance weighting (IVW), they found that *Coprococcus* abundance was negatively associated with constipation (OR = 0.74, 95% CI 0.64–0.86, *p* = 0.0001), while Bacteroidetes abundance was positively associated (OR = 1.22, 95% CI 1.00–1.50, *p* = 0.04). Reverse MR analysis did not identify significant evidence supporting a causal effect of constipation on microbiota composition. These findings provide genetic-level evidence consistent with a directional contribution of some microbiota alterations to constipation susceptibility; however, they do not establish biological causation, and the results should be interpreted with caution given the inherent limitations of MR (discussed in Section 5.1).

The MR study by He et al. (2024) (two-sample MR) reported that *Anaerotruncus* (OR = 1.08, 95% CI 1.02–1.13, *p* = 0.007), *Butyricimonas* (OR = 1.07, 95% CI 1.01–1.13, *p* = 0.015) and *Hungatella* (OR = 1.03, 95% CI 1.00–1.06, *p* = 0.037) were positively associated with constipation risk, while *Ruminiclostridium 9* (OR = 0.75, 95% CI 0.73–0.78, *p* < 0.001) and *Intestinibacter* (OR = 0.89, 95% CI 0.86–0.93, *p* < 0.001) were negatively associated.

Zheng J. et al. (2025) further explored the relationship between Parkinson’s disease, gut microbiota and constipation using MR with mediation analysis. They found that the *E. rectale* cluster and Methanobacteriales mediated the effect of Parkinson’s disease on constipation, with mediation proportions of 193.17 and 128.44%, respectively—a finding that provides new clues for understanding constipation associated with neurological diseases. A comprehensive overview of the microbiota alterations discussed in this section, including their evidence consistency and key controversies, is provided in Table 1.

**Table 1:** Evidence consistency and key controversies in microbiota alterations associated with constipation.

Category/Finding	Specific content	Key alterations/mechanisms	Evidence type	Evidence consistency	Key controversies/limitations	References
Gut microbiota	Butyrate-producing bacteria	↓*Faecalibacterium*, *Roseburia*, *Coprococcus*, *E. rectale* group	Human observational; MR evidence	High: multiple studies, MR supportive	Reverse causality cannot be fully excluded	(Zhuang et al., 2019; Han et al., 2024; Louis and Flint, 2017; Zhou et al., 2024; Tian et al., 2021)
	Potentially pathogenic bacteria	↑*Bacteroides*, *Parabacteroides*, *Desulfovibrionaceae*, Enterobacteriaceae	Human observational; MR evidence	Moderate: consistent in STC and IBS-C; MR supportive	Few studies in pure FC; species-level heterogeneity	(Fan et al., 2022; Zhou et al., 2024; Roy et al., 2025)
	Methanogens	↑*M. smithii* (observational) vs. negative correlation (MR)	Human observational; MR evidence	Low: direction differs by study type	Association may reflect prolonged transit time rather than direct causation; MR findings remain inconsistent	(Hoegenauer et al., 2022; Culqui Lévano et al., 2025; Zheng J. et al., 2025)
	*Bifidobacterium*/*Lactobacillus*	Decreased in some studies, unchanged in others	Human observational	Low: method-dependent	DNA extraction bias (bead-beating) for Gram-positive bacteria; effect may be artefactual	(He et al., 2022; Zhu et al., 2014; Lim et al., 2018; Erhardt et al., 2023)
Subtype differences	STC	↑*G. pamelaeae*, *B. longum*, *A. colihominis*; ↓*C. comes*, *R. intestinalis*; altered bile acid pathways	Human metagenomics; multi-omics	Moderate: few studies but consistent	Unknown whether changes are cause or consequence of delayed transit	(Han et al., 2024; Fan et al., 2022)
	IBS-C	↑*E. coli*, Enterobacteriaceae; ↓*Bifidobacterium*, *Lactobacillus*; ↓Butyrate-producing *Roseburia-E. rectale* group	Human observational; meta-analysis	Limited: small studies, not fully replicated	Overlap with FC and STC signatures not well characterized; geographic variation not studied	(Roy et al., 2025; Chassard et al., 2012)
	Mucosa- vs. fecal-associated	Mucosal microbiota more closely linked to symptoms; fecal correlates with transit time and methane	Human observational (colonic biopsies vs. stool)	High: consistent across multiple studies	Mechanistic link between sampling site and clinical relevance requires further exploration	(Parthasarathy et al., 2016; Quigley and Spiller, 2016)
Key metabolites	SCFAs	↓Acetate, propionate, butyrate	Animal mechanistic; human metabolomics (limited)	Strong (animal), limited human data	Human validation remains incomplete; pathway contribution in patients unclear	(Fan et al., 2022; Martin-Gallausiaux et al., 2021; Tan et al., 2014; Ge et al., 2018; Reigstad et al., 2015; Blakeney et al., 2019; Kaji et al., 2018; Soret et al., 2010; Vincent et al., 2018; Peng et al., 2009; Xu et al., 2024)
	Key mechanisms	• ↑5-HT release (GPR41/43)	—	—	—	(Ge et al., 2018; Reigstad et al., 2015)
		• ↑ENS excitability (butyrate via MCT2)	—	—	—	(Soret et al., 2010; Vincent et al., 2018)
		• ↑Smooth muscle contractility (direct action)	—	—	—	(Blakeney et al., 2019; Kaji et al., 2018)
		• ↑Barrier integrity (tight junction assembly via AMPK)	—	—	—	(Peng et al., 2009; Miao et al., 2016)
	Bile acids	↓DCA, LCA; ↓TGR5/FXR in STC colonic tissue	Human multi-omics (single study)	Moderate: mainly from one multi-omics study (Fan et al., 2022); preliminary probiotic data	Replication needed across independent cohorts; small sample size	(Fan et al., 2022; Ticho et al., 2019; Duboc et al., 2014; Zheng F. et al., 2025)
	Key mechanisms	• Activate TGR5 → ↑5-HT release → accelerated colonic transit	—	—	—	(Ticho et al., 2019; Duboc et al., 2014)
		• ↓TGR5/FXR expression in STC correlates with delayed transit	—	—	—	(Fan et al., 2022)
	Methane	↑Produced by *M. smithii*; inhibits peristalsis (animal); human associations contradictory	Animal mechanistic; human observational; MR	Low/contradictory	Observational studies show positive association, but MR suggests negative correlation; causality unclear	(Culqui Lévano et al., 2025; Triantafyllou et al., 2014; Jahng et al., 2012; Wang and Yao, 2021)
Key signaling molecules	5-HT	Core brain-gut signaling molecule (>90% synthesized by EC cells); abnormal in constipation (↓5-HT, ↑SERT)	Animal mechanistic; early human evidence	Strong (animal), limited human data	Human replication needed; therapeutic translation not yet validated	(Cheng et al., 2024; Ge et al., 2018; Zhang Y. et al., 2020; Agus et al., 2018; Mawe and Hoffman, 2013; Yano et al., 2015; Reigstad et al., 2015; Cao et al., 2017; Zhan et al., 2021; Omer and Quigley, 2017)
	TGR5/FXR	Bile acid receptors; TGR5 promotes 5-HT release; down-regulated in STC colonic tissue	Human tissue (limited)	Limited human data	Small sample size; needs larger studies	(Fan et al., 2022; Ticho et al., 2019; Duboc et al., 2014)
	c-kit/SCF	ICC marker; expression ↓ in constipation models, restored by probiotics	Animal only	Animal only	No human ICC data in constipation; translational gap	(Cheng et al., 2024; Pan et al., 2022)
	TLR2/TLR4	Pattern recognition receptors on enteric neurons; microbial products promote neuronal survival via TLR4 signaling	Animal only	Animal only	Translational relevance to human constipation unclear	(Bai et al., 2025; Anitha et al., 2012; Yarandi et al., 2020)

STC, slow-transit constipation; FC, functional constipation; IBS-C, constipation-predominant irritable bowel syndrome; MR, Mendelian randomization; SCFA, short-chain fatty acid; 5-HT, 5-hydroxytryptamine; EC cells, enterochromaffin cells; ENS, enteric nervous system; MCT2, monocarboxylate transporter 2; AMPK, AMP-activated protein kinase; DCA, deoxycholic acid; LCA, lithocholic acid; TGR5, G-protein-coupled bile acid receptor; FXR, farnesoid X receptor; SERT, serotonin transporter; ICC, interstitial cells of Cajal; SCF, stem cell factor; TLR, Toll-like receptor.

MR has important limitations, including horizontal pleiotropy, limited variance explained by genetic instruments, and potential reverse causality (see Section 5.1 for detailed discussion).

These causal inferences set the stage for understanding the mechanistic pathways through which the gut microbiota influences intestinal motility, discussed in the following section.

## 3 The BGA: a central mechanism linking microbiota and constipation

The BGA is a complex bidirectional communication network, and the gut microbiota influences intestinal motility and sensation through neural, metabolic and immune pathways (Holzer and Farzi, 2014; Zheng et al., 2022). Much of the mechanistic evidence discussed in this section is derived from animal models or ex vivo human tissue studies. Although these studies provide valuable insights into potential pathways, the extent to which each mechanism contributes to constipation pathophysiology in human patients—and whether these pathways are independent or synergistic—remains incompletely understood. Wherever possible, we indicate the nature and strength of the supporting evidence throughout this section.

### 3.1 Neural pathways

The ENS, often called the “second brain,” comprises hundreds of millions of neurons within the gastrointestinal wall and can regulate motility and secretion independently of the central nervous system (Sasselli et al., 2012). The gut microbiota plays an important role in ENS development and maintenance. A review by Bai et al. (2025) noted that germ-free mice exhibit reduced ENS neuronal density and slowed gut transit, while bacterial colonization restores ENS network density and neuronal excitability. This effect is partly mediated by Toll-like receptors (TLRs): TLR2 and TLR4 are expressed on enteric neurons, and microbial components (e.g., lipopolysaccharide) promote neuronal survival via TLR4 signaling (Anitha et al., 2012). Yarandi et al. (2020) confirmed that TLR2-mediated neurogenesis is essential for maintaining adult mouse ENS and colonic motor function.

Interstitial cells of Cajal (ICC) are specialized mesenchymal cells that function as electrical pacemakers within the gastrointestinal tract. ICC generate and propagate slow-wave electrical activity that coordinates rhythmic smooth muscle contractions and regulates intestinal peristalsis (Sanders, 2019). Accumulating evidence suggests that ICC dysfunction is an important pathophysiological feature of STC. Reduced ICC density, impaired c-kit signaling, and disrupted ICC ultrastructure have been observed in both experimental constipation models and patients with STC (Cheng et al., 2024; Pan et al., 2022). ICC are not passive target cells; they form functional syncytia with enteric neurons and smooth muscle cells through gap junctions, predominantly via connexin 43 (Cx43). This structural arrangement enables ICC to serve as intermediaries for enteric motor neurotransmission, receiving signals from neurons and conducting them to adjacent smooth muscle cells. Through these gap-junction-connected networks, ICC propagate and coordinate slow-wave activity across long distances, ensuring peristaltic coordination (Blair et al., 2014; Hanani et al., 2005). Importantly, emerging evidence suggests that the gut microbiota may indirectly influence ICC function through microbial metabolites and neuroimmune pathways (Macpherson et al., 2023). SCFAs can modulate enteric neuronal activity and intestinal smooth muscle function (Bruning et al., 2020), whereas serotonin signaling influences ICC-mediated neuromuscular coordination (Huizinga et al., 2021). Low-grade inflammation and altered macrophage activation may also impair ICC survival and pacemaker activity (Huizinga et al., 2021; De Schepper et al., 2018). Beyond their interactions with neurons and smooth muscle, ICC also engage in bidirectional crosstalk with muscularis macrophages (MMφ). In inflammatory conditions such as TNBS-induced colitis, MMφ accumulate in the ICC network and myenteric plexus, and ICC integrity is compromised. Mechanistically, MMφ regulate ICC survival and pacemaker activity through paracrine signaling. A central pathway involves bone morphogenetic protein 2 (BMP2) secreted by MMφ. BMP2 binds to BMP receptors on enteric neurons, which in turn produce CSF1, a growth factor essential for MMφ homeostasis. This MMφ-neuron-ICC axis is further modulated by microbial signals: commensal-derived stimuli (such as lipopolysaccharide acting via TLR4 on MMφ) regulate BMP2 expression, thereby influencing motility (Muller et al., 2014; Kinoshita et al., 2007). An emerging paradigm that has not yet been extensively reviewed in the constipation literature is the microbiota-macrophage-ICC axis. Dysbiosis may influence ICC function indirectly through MMφ. Commensal microbiota modulate MMφ phenotype and BMP2 production, and changes in microbial composition in constipation have been linked to MMφ activation. In a humanized mouse model, colonization with microbiota from severely constipated patients resulted in slow colonic transit, which was associated with damage to the ICC network driven by pro-inflammatory macrophages. This suggests a pathway whereby dysbiosis → MMφ activation → ICC injury → dysmotility, although direct mechanistic evidence connecting specific microbial taxa to MMφ-mediated ICC impairment remains limited, and most of the current evidence is derived from animal models (Muller et al., 2014). Further mechanistic studies integrating microbiome analysis with electrophysiological and histological assessment of ICC networks are required.

The vagus nerve is a major bidirectional pathway connecting the gut to the central nervous system. Intestinal microbiota and their metabolites can activate vagal afferent fibers, transmitting signals to brain regions such as the nucleus tractus solitarius, thereby regulating intestinal motor and secretory functions (Liu and Forsythe, 2021). Wan et al. (2025) indicated that vagotomy abolishes the modulatory effects of lactobacilli on emotional behaviors in mice, underscoring the critical role of the vagus nerve in microbiota-brain communication.

### 3.2 Metabolic pathways

#### 3.2.1 SCFAs

SCFAs (mainly acetate, propionate and butyrate) are major metabolites produced by fermentation of dietary fiber by the gut microbiota. They play a central role in constipation pathogenesis (Morrison and Preston, 2016; Koh et al., 2016).

Production and sources. Dietary fiber is fermented to SCFAs: butyrate is mainly derived from Firmicutes (e.g., *Faecalibacterium*, *Roseburia*, *Coprococcus*, *Eubacterium*), propionate mainly from Bacteroidetes and some Negativicutes, and acetate from a wide range of bacteria (Flint et al., 2012; Louis and Flint, 2017). Fecal SCFA levels are significantly lower in patients with constipation. Fan et al. found that propionate levels were significantly lower in STC patients than in controls (*p* = 0.013) (Fan et al., 2022). Lai et al. (2023) reported that baseline SCFA levels in constipated patients were positively correlated with bowel movement frequency.

Several complementary mechanisms have been proposed to explain how SCFAs may influence intestinal motility, although most evidence derives from animal studies and the relative contribution of each pathway in humans remains incompletely characterized (Martin-Gallausiaux et al., 2021; Tan et al., 2014). First, activation of enterochromaffin (EC) cells: SCFAs activate EC cells via GPR41 and GPR43, promoting 5-hydroxytryptamine (5-HT) synthesis and release, which in turn stimulates the peristaltic reflex (Ge et al., 2018; Reigstad et al., 2015). Wan et al. (2025) emphasized that 5-HT is a core signaling molecule in the BGA, and SCFAs promote 5-HT synthesis by up-regulating tryptophan hydroxylase 1 (TPH1) expression. Second, direct action on smooth muscle: SCFAs can directly stimulate colonic smooth muscle contraction, an effect insensitive to tetrodotoxin, suggesting it is not neuron-dependent (Blakeney et al., 2019; Kaji et al., 2018). Third, modulation of ENS neuronal phenotype: butyrate enhances the excitability of choline acetyltransferase (ChAT)-positive neurons via monocarboxylate transporter 2 (MCT2), promoting colonic transit (Soret et al., 2010; Vincent et al., 2018). Fourth, maintenance of intestinal barrier function: butyrate enhances tight junction assembly through activation of AMP-activated protein kinase (AMPK) (Peng et al., 2009; Miao et al., 2016). Xu et al. (2024) summarized that SCFAs maintain barrier integrity by stimulating tight junction protein expression and mucin-related peptide production.

#### 3.2.2 Bile acids (BAs)

BAs are not only emulsifiers essential for lipid digestion and absorption, but also signaling molecules with hormone-like effects, regulating energy metabolism, intestinal integrity and immune function through nuclear (farnesoid X receptor, FXR) and membrane (G-protein-coupled bile acid receptor, TGR5) receptors (Fiorucci and Distrutti, 2015; Wahlström et al., 2016).

Enterohepatic circulation and microbial transformation. Primary BAs (cholic acid, chenodeoxycholic acid) are synthesized in hepatocytes from cholesterol and secreted into the intestine. Approximately 95% are reabsorbed at the terminal ileum; the remaining 5% enter the colon, where they undergo deconjugation and transformation by the gut microbiota to produce secondary BAs (deoxycholic acid DCA, lithocholic acid LCA, etc.) (Ridlon et al., 2006; Staley et al., 2017). Key microbial genera involved include *Bacteroides*, *Clostridium* and *Eubacterium* (Winston and Theriot, 2020).

Mechanisms of bile acid regulation of intestinal motility. Secondary BAs activate TGR5 receptors on EC cells, promoting 5-HT release and accelerating colonic transit (Ticho et al., 2019; Duboc et al., 2014). Fan et al. (2022) found significant down-regulation of TGR5 and FXR expression in colonic tissues of STC patients, which correlated with delayed transit. Additionally, chenodeoxycholic acid promotes chloride and water secretion into the intestinal lumen, softening feces, by activating adenylate cyclase and increasing intracellular cAMP (Hegyi et al., 2018; Ao et al., 2013).

Abnormal bile acid metabolism in constipation. A multi-omics analysis by Fan et al. (2022) showed significantly lower levels of several BAs in feces of STC patients. A randomized clinical trial by Zheng F. et al. (2025) (recent conference abstract) reported that *Lactiplantibacillus plantarum* Probio87 intervention increased bile acid levels by 38%, which correlated with improved bowel movement frequency (*r* = 0.85). However, as these data are preliminary, they require confirmation in fully published peer-reviewed studies.

#### 3.2.3 Methane

Methane is produced by intestinal methanogenic archaea, mainly *M. smithii*, and methane production has frequently been associated with constipation (Triantafyllou et al., 2014). In the study by Culqui Lévano et al. (2025), constipated patients receiving synbiotic supplementation showed a reduction in *M. smithii* accompanied by improved stool consistency. Animal experiments have shown that methane reduces intestinal peristalsis and prolongs colonic transit time (Jahng et al., 2012). Wang and Yao (2021) suggested that methane may act as a neuromuscular transmitter, affecting 5-HT levels and intestinal neuromuscular function; however, direct experimental validation in human tissues is still limited, and as discussed in Section 2.2, the direction of association is not consistent across studies, warranting further investigation.

### 3.3 Immune pathways

Intestinal barrier dysfunction is an important feature in patients with constipation. Khalif et al. (2005) found elevated serum ovalbumin concentrations in patients with chronic constipation, suggesting increased intestinal permeability. Pan et al. (2022) indicated that constipation-induced dysbiosis may lead to decreased mucin (MUC2) expression, thinning of the mucus layer, and damage to the epithelial barrier. Xu et al. (2024) confirmed that probiotics can improve barrier function by regulating tight junction protein expression and enhancing mucus secretion.

Low-grade inflammation plays a role in constipation pathogenesis. Constipated patients exhibit increased T-cell activation and enhanced lymphocyte proliferation (Khalif et al., 2005). Bai et al. (2025) indicated that inflammation-induced CD8^+^ T-cell adhesion to enteric neurons can cause acute neuronal injury, colonic dilatation and slowed transit. SCFAs may attenuate inflammatory responses by modulating Treg cell populations and function (Liu et al., 2023).

MMφ have recently been identified as regulators of intestinal motility. Bai et al. (2025) detailed the interaction of MMφ with ICC and mesenchymal stromal cells. Cogo et al. (2025) demonstrated that MMφ interact with intestinal neurons through production of BMP2 to regulate motility. Antibiotic treatment reduces MMφ numbers, leading to impaired motility, whereas lipopolysaccharide supplementation increases MMφ numbers and improves motility (Becker et al., 2019).

### 3.4 The central role of 5-HT

5-HT is a central signaling molecule in the BGA; more than 90% of 5-HT is synthesized in gastrointestinal EC cells (Zhang Y. et al., 2020; Guzel and Mirowska-Guzel, 2022).

Synthesis and metabolism. Tryptophan is converted to 5-hydroxytryptophan by TPH1, then decarboxylated by aromatic amino acid decarboxylases to produce 5-HT (Agus et al., 2018). 5-HT acts through more than 14 receptor subtypes; the 5-HT_3_ and 5-HT_4_ receptors play key roles in intestinal motility (Guzel and Mirowska-Guzel, 2022; Najjar et al., 2023; Mawe and Hoffman, 2013). 5-HT reuptake is mediated by the serotonin transporter (SERT) (Mawe and Hoffman, 2013).

Modulation of 5-HT signaling by gut microbiota. Several studies have shown that the gut microbiota regulates 5-HT synthesis. Yano et al. (2015) found that indigenous spore-forming bacteria promote 5-HT production by EC cells. Reigstad et al. (2015) showed that germ-free mice have reduced TPH1 expression and decreased colonic 5-HT levels, which are restored after bacterial colonization. Wan et al. (2025) detailed the mechanism by which SCFAs promote 5-HT release through activation of FFAR2/3 receptors on EC cells.

Abnormal 5-HT signaling in constipation. 5-HT signaling abnormalities are present in constipated patients. Cao et al. (2017) found that 5-HT levels were reduced and SERT expression elevated in the colon of germ-free mice transplanted with microbiota from constipated patients—a finding that, while compelling, requires replication in human studies before broader conclusions can be drawn. Cheng et al. (2024) showed that constipated mice had reduced serum 5-HT and increased NO levels, and probiotic interventions reversed these alterations. Zhan et al. (2021) showed that paeoniflorin promotes 5-HT secretion via the TGR5/TRPA1 signaling pathway and ameliorates loperamide-induced constipation.

5-HT_4_ receptor as a therapeutic target. 5-HT_4_ receptor agonists (e.g., prucalopride) promote acetylcholine release and enhance colonic propulsive motility, and are used clinically for chronic constipation (Omer and Quigley, 2017). Probiotics may up-regulate 5-HT_4_ receptor expression; Cheng et al. (2024) reported that probiotic-fermented milk up-regulated colonic 5-HT_4_ receptor mRNA in mice. Thus, targeting the 5-HT signaling pathway, particularly up-regulating 5-HT_4_ receptor expression through probiotics, represents one potential strategy for constipation treatment (discussed in Section 4). However, clinical evidence supporting this mechanism in humans is still preliminary, and the extent to which probiotic-induced 5-HT modulation translates into meaningful symptom improvement remains to be established.

**Figure 1:**
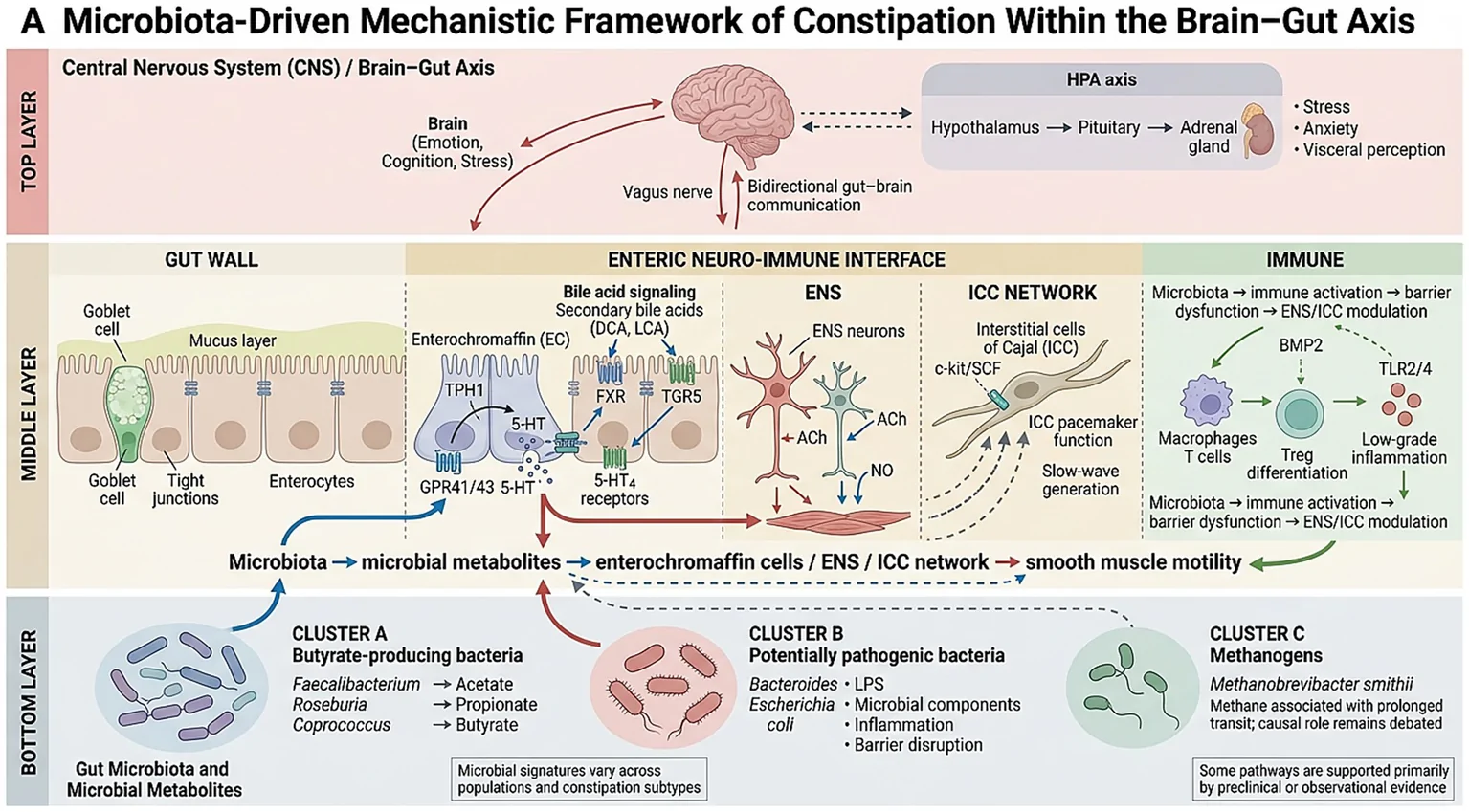
A microbiota-driven mechanistic framework of constipation within the brain-gut axis.

The complex interactions between the gut microbiota and host via the BGA are summarized in Figure 1. Figure 1 integrates neural (ENS, ICC, vagus), metabolic (SCFAs, BAs, methane) and immune pathways involved in the regulation of intestinal motility.

## 4 Microbiota-based intervention strategies: clinical evidence

### 4.1 Probiotics

#### 4.1.1 Definition

Probiotics are live microorganisms that confer a health benefit when administered in adequate amounts (Hill et al., 2014).

#### 4.1.2 Mechanisms of action

Probiotics may improve constipation through multiple mechanisms: (1) producing SCFAs (particularly butyrate) through fermentation of dietary fiber; (2) enhancing intestinal barrier function by up-regulating tight junction proteins; (3) modulating gut transit via 5-HT and bile acid signaling; (4) suppressing pro-inflammatory pathways; and (5) competing with pathogenic bacteria for adhesion sites and nutrients.

Numerous RCTs and meta-analyses have assessed the efficacy of probiotics for constipation. However, the evidence is limited by substantial heterogeneity and generally low-to-moderate quality, and results should be interpreted cautiously.

#### 4.1.3 Evidence synthesis

Multiple meta-analyses suggest that probiotics may modestly improve bowel frequency and stool consistency in some patients with FC; however, the overall certainty of this evidence remains low due to marked heterogeneity in strains, dosages, treatment durations, and outcome definitions. For example, the largest meta-analysis to date, by Ding et al. (2024) (17 RCTs, 1,256 patients), reported that probiotics significantly increased bowel movement frequency (WMD 0.93, 95% CI 0.47–1.40, *p* = 0.000) and modestly improved stool consistency (WMD 0.38, 95% CI 0.05–0.70, *p* = 0.023). A separate meta-analysis by Zhang C. et al. (2020) (15 RCTs, 934 adults) similarly concluded that probiotics improved constipation symptoms. However, GRADE grading in the Ding et al. (2024) meta-analysis rated the quality of evidence as “low” for bowel frequency and “very low” for stool consistency and PAC-SYM, primarily because of high heterogeneity across studies and limited trial numbers. Thus, while meta-analytic estimates suggest small beneficial effects, these estimates are based on low-certainty evidence and should be interpreted cautiously.

#### 4.1.4 Strain-specific effects

A randomized, double-blind, placebo-controlled trial by Takeda et al. (2023) (80 elderly patients with chronic constipation) administered *B. longum* BB536 (5 × 10^10^ CFU/day) for 4 weeks. The primary endpoint (total CSS score) did not reach statistical significance (*p* = 0.074), representing moderate-quality evidence that does not support a conclusive benefit; however, exploratory subgroup analyses suggested improvements in bowel frequency (*p* = 0.008) and sense of incomplete evacuation (*p* = 0.051). The study supports the safety of BB536 in elderly patients, but the negative primary endpoint warrants caution in interpretation.

#### 4.1.5 *Lactobacillus reuteri* DSM 17938

An RCT by Ojetti et al. (2014) showed that a 4-week intervention with *L. reuteri* DSM 17938 significantly increased bowel movement frequency in adults with FC. Positive results were also obtained in infants (Coccorullo et al., 2010) and in reducing abdominal pain in children (Jadrešin et al., 2020). However, most studies are small and short-term.

#### 4.1.6 *L. plantarum* Probio87

A conference abstract (IDDF2025) reported preliminary findings from a multi-omics RCT in 101 patients with FC (12-week intervention), suggesting that Probio87 may increase *L. plantarum* abundance and improve CSBM frequency relative to placebo (Zheng F. et al., 2025). However, as these data have not yet undergone full peer review, the reported effect sizes (week 12 ΔCSBM +1.21 vs. +0.42, *p* = 0.00015; 100% of Probio87-treated patients achieved CSBM ≥3/week vs. 64% of placebo recipients) and mechanistic interpretations (bile acid-driven motility) require independent replication and should be interpreted with substantial caution. These findings are hypothesis-generating and do not constitute evidence for clinical efficacy (Zheng F. et al., 2025).

#### 4.1.7 Multi-strain probiotics

Moderate-certainty evidence from meta-analyses (e.g., Ding et al., 2024) suggests that multi-strain preparations may be more effective than single strains (Ding et al., 2024). Supportive, lower-quality evidence comes from a small clinical trial by Luo et al. (2025), in which 20 STC patients receiving a triple-viable probiotic (*B. longum*, *L. bulgaricus*, *S. thermophilus*) for 4 weeks showed faster gastrointestinal transit time (*p* = 0.012) and improved CSS scores. Preliminary evidence from animal studies (He et al., 2022) further indicated that a five-strain probiotic mixture significantly improved intestinal transit rate and stool consistency in constipated mice (He et al., 2022); however, animal findings do not directly translate to human efficacy and require human validation.

#### 4.1.8 Probiotic colonization

He et al. (2022) assessed colonization using absolute quantification. *L. acidophilus* increased only during intervention and returned to baseline after discontinuation, whereas *L. plantarum* and *L. rhamnosus* remained at high abundance for 2 weeks after discontinuation, indicating successful colonization. Higher doses promoted *L. rhamnosus* colonization, while lower doses favored *L. plantarum* colonization. These findings suggest that colonization capacity varies among strains and may influence efficacy.

#### 4.1.9 Limitations

The main limitations of probiotic studies include high heterogeneity (*I*^2^ = 81–85%), small trial numbers, varied outcome definitions, short treatment durations, and potential publication bias.

#### 4.1.10 Summary

Certain probiotic strains may improve bowel frequency and stool consistency in selected patients with constipation; however, clinical efficacy appears strain-specific and overall evidence quality remains low to moderate. Although several meta-analyses have reported modest benefits of probiotics in constipation, the certainty of evidence remains low to very low because of substantial heterogeneity, small sample sizes, inconsistent endpoints, and potential publication bias. Among the available low-certainty evidence, selected *Bifidobacterium* and *Lactobacillus* strains have shown relatively more consistent results; however, many studies involve short treatment durations and lack mechanistic validation. These findings should be interpreted as hypothesis-generating rather than practice-changing.

### 4.2 Prebiotics

#### 4.2.1 Definition

Prebiotics are substrates selectively utilized by host microorganisms conferring a health benefit (Gibson et al., 2017). Common prebiotics include inulin, fructo-oligosaccharides (FOS), galacto-oligosaccharides (GOS) and lactulose (Davani-Davari et al., 2019).

#### 4.2.2 Mechanisms of action

Prebiotics selectively stimulate the growth or activity of beneficial bacteria, particularly *Bifidobacterium* and *Lactobacillus* species, thereby increasing SCFA production, improving intestinal barrier function, and enhancing gut motility (Gibson et al., 2017).

#### 4.2.3 Evidence

Moderate-quality evidence from a well-designed randomized, double-blind, placebo-controlled crossover trial by Puhlmann et al. (2025) (39 adults with FC) used 12 g/day inulin or placebo (maltodextrin) for 4 weeks. Inulin increased bowel frequency by 1.43 movements/week (placebo: 0.90; between-group difference *p* = 0.046). PAC-QOL total score improvement was significantly better with inulin (−0.61 vs. -0.21, *p* = 0.007), particularly in psychosocial discomfort and worry domains. The relative abundance of butyrate-producing *Anaerostipes* and *Coprococcus 1* increased, and *Coprococcus 1* abundance correlated positively with bowel frequency (*ρ* = 0.40, *p* = 0.02). A notable carryover effect was observed: subjects receiving inulin first maintained partially altered microbiota and clinical benefits beyond the 4-week washout. Baseline microbiota characteristics (higher butyrate producers, lower *Bifidobacterium*) predicted response, suggesting potential for personalized prebiotic interventions.

Next, Dext™ (dextran prebiotic). Low-certainty evidence from a small randomized, double-blind, parallel-controlled trial by Culqui Lévano et al. (2025) (24 constipated patients, 2-week intervention) compared NextDext™ (5 g, three times daily) with ABB C24 (NextDext™ + postbiotic yeast). The NextDext™ group showed improvement in Bristol stool score from 2.33 to 3.50 (*p* = 0.064, trend), close to normal (Drossman and Hasler, 2016; Sperber et al., 2021). Notably, 90% of patients used opioids, and NextDext™ restored opioid-associated dysbiosis (increased *B. longum* and *Roseburia hominis*, decreased *Blautia obeum*, *E. coli*, *Collinsella aerofaciens*). These preliminary findings suggest potential utility in opioid-induced constipation, but the small sample size and short duration limit generalizability, and larger confirmatory studies are needed.

#### 4.2.4 Limitations

The main limitations of prebiotic studies include a single well-designed trial for inulin, carryover effects suggesting potential period-related bias, and very small sample size for NextDext™.

#### 4.2.5 Baseline microbiota predicts response

The Puhlmann et al. (2025) study showed that responders (those with carryover effects) had higher baseline abundance of *Faecalibacterium* and *Roseburia* and lower *Bifidobacterium*. This aligns with earlier findings by de Preter et al. (2008) that baseline *Bifidobacterium* counts influence prebiotic response, supporting the concept of personalized prebiotic selection.

### 4.3 Synbiotics

#### 4.3.1 Definition

Synbiotics are mixtures of probiotics and prebiotics that work synergistically (Swanson et al., 2020).

#### 4.3.2 Mechanisms of action

Synbiotics combine the beneficial effects of probiotics (live microbial supplementation) and prebiotics (substrate for beneficial bacteria) to synergistically enhance microbial engraftment, metabolite production, and clinical efficacy (Swanson et al., 2020).

#### 4.3.3 Precise pairing based on generation time

Wang et al. (2026) proposed a synbiotic pairing strategy based on bacterial generation time (GT) on different oligosaccharides. In a loperamide-induced constipation mouse model, GT-optimized synbiotics outperformed single probiotics or prebiotics alone (reduced first black stool time, increased fecal water content). Mechanistic studies showed restoration of colonic neurotransmitter homeostasis, inhibition of aquaporin expression, enrichment of butyrate-producing bacteria, and repair of intestinal barrier.

#### 4.3.4 Clinical evidence

The meta-analysis by Ding et al. (2024) suggested that synbiotics are more effective than probiotics alone for increasing bowel frequency. An RCT by Ding et al. (2016) in STC patients (12-week synbiotic containing probiotics and pectin) significantly improved constipation symptoms. An RCT by Kim et al. (2021) demonstrated that a synbiotic formulation containing six probiotic strains and xylo-oligosaccharides improved defecation-related discomfort in FC.

#### 4.3.5 Limitations

Most studies are small and heterogeneous; few dedicated RCTs; heterogeneous formulations; animal data require human validation.

#### 4.3.6 Summary

Synbiotics may offer broader applicability than probiotics alone, but more data are needed.

### 4.4 Postbiotics

#### 4.4.1 Definition

Postbiotics are preparations of inanimate microorganisms and/or their components that confer a health benefit (Salminen et al., 2021). They offer safety and stability advantages.

#### 4.4.2 Mechanisms of action

Postbiotics exert bioactivity through microbial components (e.g., cell wall fragments, exopolysaccharides) or metabolites (e.g., SCFAs, enzymes) without requiring live bacteria, thereby reducing the risk of infection or translocation in immunocompromised patients (Salminen et al., 2021).

#### 4.4.3 Evidence

ABB C24 (NextDext™ + heat-inactivated yeast). In the study by Culqui Lévano et al. (2025), a 2-week intervention with ABB C24 improved Bristol score from 2.33 to 2.75. ABB C24 decreased *B. obeum* and *M. smithii* and increased *Clostridium leptum* and *Coprococcus* spp. It also increased microbial diversity in a greater proportion of subjects than NextDext™ alone (richness index: 75% vs. 50%; Shannon index: 58% vs. 17%). Although NextDext™ modulated more species (19 vs. 9), the species modulated by ABB C24 (e.g., reduction in *M. smithii*, increase in *C. leptum* and *Coprococcus*) aligned more closely with constipation-associated alterations, suggesting additional value of postbiotic yeast.

#### 4.4.4 Heat-inactivated probiotics

A multicenter RCT by Andresen et al. (2020) showed that heat-inactivated *Bifidobacterium bifidum* MIMBb75 effectively ameliorated IBS symptoms regardless of subtype. Marasco et al. (2024) also mentioned promising use of postbiotics in disorders of gut-brain interaction. Although these studies are in IBS rather than pure constipation, they suggest that postbiotics may be a viable alternative for patients who cannot tolerate live probiotics.

#### 4.4.5 Comparative evidence across postbiotic types

The current evidence on postbiotics for constipation is derived predominantly from a limited number of formulations and study populations. The ABB C24 study (heat-inactivated yeast combined with a dextran prebiotic) focused specifically on opioid-induced constipation in a small cohort (Culqui Lévano et al., 2025), while the heat-inactivated *B. bifidum* MIMBb75 trial was conducted in IBS rather than pure constipation populations (Andresen et al., 2020). Beyond these, several other postbiotic formulations have been investigated in constipation or constipation-related settings. Heat-inactivated *Lactobacillus gasseri* CP2305 has been shown to improve bowel function and gut microbiota diversity in generally healthy adults, with particular benefit observed in populations with constipation tendencies (Sugawara et al., 2016). Heat-treated *Lactobacillus helveticus* CP790-fermented milk was evaluated in a randomized, double-blind, placebo-controlled trial in healthy Japanese adults with a tendency toward constipation; the results suggested that CP790-fermented milk could modulate gut microbiota and improve constipation symptoms (Tanihiro et al., 2024). Yeast-based postbiotics have also shown promise: the *Saccharomyces cerevisiae*-derived postbiotic EpiCor has been shown in clinical studies to reduce intestinal symptoms in constipated populations and to support mucosal defense, with *in vitro* evidence suggesting anti-inflammatory potential and butyrogenic properties (Duysburgh et al., 2024).

However, no head-to-head comparisons have been published directly comparing different categories of postbiotics—such as heat-killed whole bacterial cells (*Lactobacillus* vs. *Bifidobacterium* vs. yeast-based formulations), bacterial lysates, purified microbial metabolites (e.g., SCFAs, exopolysaccharides), or cell wall components—for constipation outcomes in the same study population. Consequently, it remains unknown whether certain postbiotic types are more effective than others for specific constipation phenotypes, and whether the metabolic or microbial signatures of the host microbiome influence postbiotic responsiveness. This represents a priority area for future comparative and mechanistic studies.

#### 4.4.6 Advantages and limitations of postbiotics relative to live probiotics

Postbiotics offer several distinctive advantages over live probiotics that are particularly relevant for specific patient populations. First, safety in immunocompromised and vulnerable patients—because postbiotics contain no live organisms, they carry no risk of bacteraemia, translocation, or systemic infection, which are concerns associated with live probiotic administration in severely immunosuppressed, critically ill, or pediatric populations. Serious probiotic-related adverse effects, including bloodstream infection and sepsis, have been reported in these vulnerable groups, driving increased interest in developing non-viable bacterial products that may confer benefits without the risks of live microorganisms. This safety advantage makes postbiotics particularly suitable for patients who are immunocompromised, have compromised intestinal barrier function, or are receiving intensive care (Ranjbar et al., 2026). Second, stability and shelf-life—postbiotics are less susceptible to environmental factors such as temperature, oxygen, and gastric acid, facilitating standardization, long-term storage, and consistent dosing (Vinderola et al., 2022). Third, reduced batch-to-batch variability—manufacturing processes can be more tightly controlled for inactivated preparations than for live cultures, potentially improving product consistency (Vinderola et al., 2022). Fourth, absence of colonization requirement—postbiotic efficacy does not depend on the ability of the administered organism to colonize the host gut, which is particularly relevant given the variable and generally poor colonization of probiotics observed in clinical studies (Ranjbar et al., 2026). Fifth, bifidogenic effects—certain postbiotic preparations, such as heat-treated *Lactobacillus* LB, have been shown to stimulate the growth of beneficial bifidobacteria in human fecal communities, suggesting that postbiotics can exert prebiotic-like effects in addition to their direct bioactivity (Warda et al., 2021).

However, postbiotics also have inherent limitations relative to live probiotics. First, restricted therapeutic targets—unlike live probiotics, which can dynamically adapt to the host environment, colonize mucosal surfaces, and produce a range of metabolites *in situ* in response to local conditions, postbiotics deliver a fixed set of bioactive components with a predetermined composition and concentration. Second, unknown optimal composition—the ideal combination of microbial components, metabolites, and bioactive molecules for constipation remains undefined, and it is unclear whether whole inactivated cells, purified metabolites, or cell wall components are most effective. Third, shorter duration of effect—because postbiotics do not establish a persistent microbial presence, their effects may be more transient and may require continuous administration to maintain clinical benefit. Fourth, limited evidence base—compared with live probiotics, postbiotics have been far less studied for constipation, with the available evidence restricted to small, short-term studies or indirect evidence from IBS populations. Fifth, mechanistic uncertainty—the precise mechanisms through which different postbiotic preparations exert their effects remain incompletely characterized, and it is unclear whether benefits are mediated through direct epithelial signaling, immune modulation, or indirect effects on the resident microbiota (Ranjbar et al., 2026).

These considerations suggest that postbiotics should currently be viewed as an exploratory therapeutic option, particularly for patients who cannot tolerate live probiotics (e.g., immunocompromised individuals, critically ill patients, or those with severe barrier dysfunction), rather than a replacement for probiotic therapy in the general population. Future research should prioritize large, well-designed RCTs directly comparing different postbiotic formulations in well-characterized constipation populations, with particular attention to identifying optimal compositions, dosing regimens, and patient subgroups most likely to benefit.

#### 4.4.7 Limitations

Very small sample size for pure constipation evidence; IBS data not directly applicable.

#### 4.4.8 Summary

Postbiotics represent an exploratory option for patients who cannot tolerate live probiotics.

### 4.5 FMT

#### 4.5.1 Definition

FMT aims to re-establish normal gut microbiota by transplanting fecal microbiota from a healthy donor into a patient‘s intestine (Barbara and Ianiro, 2020).

#### 4.5.2 Mechanisms of action

FMT restores microbial diversity and function by introducing a diverse community of commensal bacteria, which can outcompete pathogenic species, restore SCFA production, strengthen intestinal barrier function, and modulate immune responses (Barbara and Ianiro, 2020).

#### 4.5.3 Evidence of efficacy

Low- to moderate-certainty evidence from a systematic review and meta-analysis by Wang et al. (2025) (9 studies, 245 patients) reported a pooled clinical remission rate of 50.7% (95% CI 38.7–62.7%) and clinical improvement rate of 64.8% (95% CI 51.4–76.3%). FMT significantly improved stool consistency (Bristol score MD = 1.32, 95% CI 1.05–1.35), quality of life (GIQLI score MD = 32.19, 95% CI 17.15–47.23), and reduced symptom severity (Wexner score MD = −4.83, 95% CI –7.15 to −2.51). Microbiota analysis showed increased beneficial bacteria (e.g., *Bifidobacterium*, *Prevotella*) and decreased pro-inflammatory Enterobacteriaceae. Supportive evidence from a network meta-analysis by Tan S. et al. (2025) (29 RCTs, 4,389 patients with FC) ranked FMT first in improving spontaneous bowel movements (SBM), complete SBM, and Bristol stool form score among non-pharmacological interventions. An umbrella review by Li et al. (2022) (7 meta-analyses) also confirmed positive efficacy of FMT for FC. However, because most individual studies included in these meta-analyses are small and have short follow-up durations, the overall evidence quality remains low to moderate.

#### 4.5.4 Long-term safety

Long-term safety data for FMT in constipation remain limited, but accumulating evidence from both prospective studies and systematic reviews suggests a favorable safety profile. A prospective study from the Hong Kong FMT Registry (123 patients, median follow-up 30.3 months, range 1–57.9 months) reported no serious adverse events definitely or probably related to FMT; new chronic conditions were identified in some patients, but causality could not be established due to underlying comorbidities (Yau et al., 2024). In a prospective study of 52 patients with STC who received FMT and were followed for 6 months, no treatment-related severe adverse events were reported, and most adverse events were self-limiting gastrointestinal symptoms (Ding et al., 2018). A large systematic review of FMT-related adverse events from 2000 to 2020 (encompassing over 10,000 FMT procedures) reported that the most frequently observed adverse events were diarrhea (10%) and abdominal discomfort/pain/cramping (7%), with serious adverse events occurring in 1.4% of patients (0.99% microbiota-related) (Marcella et al., 2021). A retrospective study in 74 children with dysbiosis-related disorders (median follow-up 12 months) confirmed that FMT was safe and effective, although long-term efficacy decreased over time (Zou et al., 2022). Nevertheless, most long-term safety data for FMT come from studies in recurrent *Clostridioides difficile* infection rather than pure FC, and prospective studies with standardized protocols and extended follow-up (≥2 years) in constipation populations are still needed to fully characterize the long-term safety profile of FMT in this specific indication.

#### 4.5.5 Real-world clinical barriers to FMT implementation

Despite promising efficacy signals from meta-analyses, the translation of FMT into routine clinical practice for constipation faces several substantial barriers. First, current clinical guidelines have not endorsed FMT for constipation. The European Society of Neurogastroenterology and Motility (ESNM) guidelines on functional constipation in adults do not recommend FMT for routine clinical use, reflecting the absence of large, high-quality RCTs with long-term follow-up (Serra et al., 2020). The American Gastroenterological Association (AGA)-American College of Gastroenterology (ACG) clinical practice guideline on chronic idiopathic constipation focuses on pharmacological agents and does not include FMT as a recommended therapy (Chang et al., 2023). Second, regulatory frameworks for FMT remain inconsistent: the FDA classifies FMT as an investigational new drug (IND) requiring regulatory approval, while other jurisdictions regulate it as a therapeutic intervention, creating inconsistencies that complicate multi-center research and clinical adoption (Thanush and Venkatesh, 2023). Third, standardized donor screening protocols for constipation indications have not been established. Current screening practices vary widely across centers, and the optimal donor microbiome composition for constipation has not been defined. Comprehensive screening typically results in only a small proportion of candidates being eligible as donors, and such screening is associated with substantial costs (He et al., 2021). Fourth, patient acceptability and logistical barriers—including the invasiveness of administration routes, the need for repeated administrations, limited reimbursement, and restricted access to qualified centers—further constrain widespread adoption. Together, these barriers explain why FMT remains largely confined to research settings for constipation.

#### 4.5.6 Distinction between adult and pediatric populations

The evidence base for FMT in constipation differs substantially between adults and children. In adult populations, most evidence comes from STC and refractory FC, with a pooled clinical remission rate of approximately 50.7% and improvement rate of 64.8% (Wang et al., 2025). Long-term safety data in adults, while accumulating, remain limited; the Hong Kong FMT Registry (123 patients, median follow-up 30.3 months) reported no serious adverse events definitely or probably related to FMT (Yau et al., 2024). In contrast, pediatric FMT data for constipation are considerably more limited. A recent randomized, double-blind, controlled trial (110 children with intractable constipation) demonstrated that retrograde colonic enema-based FMT was safe and effective, with all adverse events being mild and self-limiting (Gu et al., 2024). However, a systematic review identified only seven publications reporting FMT in pediatric patients (total 11 treated children), of whom only 3 had chronic constipation (Sha et al., 2014). Moreover, the pathophysiology of constipation in children differs substantially from that in adults—with higher prevalence of withholding behavior and functional fecal retention—which may influence FMT responsiveness. Therefore, adult FMT efficacy and safety data cannot be directly extrapolated to children. Pediatric FMT for constipation should be restricted to well-designed clinical trials with rigorous ethical oversight and age-appropriate endpoints.

#### 4.5.7 Mechanistic studies

A conference abstract (IDDF2025) reported preliminary findings from an RCT in 60 STC patients, suggesting potential benefits of FMT (Tan W. et al. 2025). However, because these data have not yet undergone peer review, the reported cure rate (45% vs. 15% in placebo) and improvement rate (80% vs. 30%) should not be considered in the overall evidence synthesis and are mentioned only for completeness. The accompanying microbiota and metabolomic findings also require independent validation before any conclusions can be drawn (Tan W. et al., 2025).

#### 4.5.8 Subgroup analysis in SIBO

Wang et al. (2024) (218 patients with chronic constipation) found that patients with coexisting small intestinal bacterial overgrowth (SIBO) responded better to FMT than those without SIBO, with significant improvements in defecation frequency, stool consistency, abdominal symptoms and quality of life (*p* < 0.05).

#### 4.5.9 Safety and limitations

The meta-analysis by Wang et al. (2025) reported that FMT-related adverse events were mainly self-limiting gastrointestinal reactions (e.g., abdominal distension, incidence 17.3%), with no serious adverse events reported. The network meta-analysis by Tan S. et al. (2025) also confirmed a good safety profile. However, long-term safety data are still lacking, and most evidence comes from small, short-term studies with heterogeneous protocols.

#### 4.5.10 Summary

FMT has shown promising results in meta-analyses; however, since most current studies are small in scale, of short duration, and vary in methodology, it should still be considered a subject of further research regarding constipation. Importantly, FMT for chronic constipation remains an investigational therapy. Current evidence is limited by small sample sizes, heterogeneous protocols, and short follow-up durations. FMT should therefore be performed only within clinical trial settings or at qualified centers with appropriate ethical oversight and informed consent.

### 4.6 Evaluation of quality of evidence and sources of heterogeneity

#### 4.6.1 GRADE grading

The meta-analysis by Ding et al. (2024) graded the quality of evidence as “low” for bowel frequency improvement, “very low” for stool consistency and PAC-SYM improvement, due to moderate-to-high heterogeneity and limited trial numbers. Overall, across all intervention categories, the highest available evidence quality is moderate (for inulin from a single well-designed RCT), while most probiotic, synbiotic, and postbiotic evidence is low to very low, and FMT evidence is low to moderate.

#### 4.6.2 Sources of heterogeneity

Heterogeneity in probiotic studies arises from strain specificity, dosage and regimen differences, population characteristics, diagnostic criteria, outcome measures, and publication bias (Ding et al., 2024; Marasco et al., 2024; Ford et al., 2018). These issues are further discussed in Section 5.1.

Table 2 provides a comparative overview of the key clinical evidence for microbiota-based interventions, organized by evidence strength (Tier 1: established/stronger evidence; Tier 2: preliminary/exploratory evidence). For each intervention, we report effect sizes, study limitations, and translational status to help readers assess the current readiness for clinical application. A separate comparison focusing on clinical applicability—including recommendation grades, reference dosages, and follow-up durations—is provided in Table 4 (Section 4.7).

**Table 2:** Current evidence and translational limitations of microbiota-targeted therapies for constipation.

Intervention	Representative agent(s) / source	Effect summary (bowel frequency / remission)	Other reported outcomes	Study design (highest available)	Evidence quality (GRADE / adapted)	Main limitations	Translational status	Key references
Tier 1: established/stronger evidence								
Probiotics (meta-analysis)	Multiple strains, predominantly *Bifidobacterium* and *Lactobacillus*	Modest improvement: WMD + 0.93 bowel movements/week (95% CI 0.47–1.40)	Improved stool consistency (WMD 0.38); ↓PAC-SYM (WMD -0.28)	Meta-analysis of 17 RCTs (*n* = 1,256)	Low to very low (GRADE: low for frequency, very low for stool consistency and PAC-SYM)	High heterogeneity (*I*^2^ = 81–85%), small trial numbers, varied outcomes	Adjunctive only (not first-line)	(Ding et al., 2024)
Prebiotics (inulin)	12 g/day inulin, 4 weeks	Modest improvement: +1.43 movements/week (95% CI not reported; *p* = 0.046)	↑PAC-QOL (−0.61 vs. −0.21, *p* = 0.007); carry-over effect observed	Single well-designed crossover RCT (*n* = 39)	Moderate (single well-designed RCT, moderate sample size)	Single study only; carry-over effect may indicate period-related bias	Adjunctive only (needs replication)	(Puhlmann et al., 2025)
FMT (meta-analysis)	Healthy donor FMT	50.7% remission rate (95% CI 38.7–62.7%); 64.8% improvement rate	↑Bristol score MD 1.32; ↑GIQLI MD 32.19; ↓Wexner score MD −4.83	Meta-analysis of 9 studies (*n* = 245 patients)	Low to moderate (9 studies, high heterogeneity, small sample sizes)	High heterogeneity, small sample sizes, short follow-up, variable protocols	Investigational (research setting only)	(Wang et al., 2025)
Tier 2: preliminary/exploratory evidence (including conference abstracts, animal studies, small uncontrolled trials)								
FMT (network meta-analysis)	Healthy donor FMT (comparator: other non-pharmacological interventions)	Ranked #1 for improving SBM, CSBM, and BSFS among non-pharmacological interventions (no direct effect size)	No direct comparison effect size reported	Network meta-analysis (29 RCTs, *n* = 4,389)	Low (based on low-quality primary studies)	Quality of individual studies generally low to moderate; network meta-analysis relies on indirect comparisons	Investigational (research setting only)	(Tan S. et al., 2025)
Probiotics (single strain, BB536)	*B. longum* BB536, 5 × 10^10^ CFU/day, 4 weeks	Primary endpoint negative; exploratory subgroup analysis suggested improvement	Improved upper GI symptoms (heartburn, dysphagia)	Single RCT (*n* = 80, elderly)	Moderate (well-designed RCT, but primary endpoint negative)	Negative primary endpoint; subgroup findings require confirmation	Adjunctive only (elderly patients)	(Takeda et al., 2023)
Probiotics (single strain, *L. reuteri* DSM 17938)	*L. reuteri* DSM 17938, 4 weeks	~+2.4 bowel movements/week in children; significant increase in adults	Reduced abdominal pain in children (additional benefit)	Small RCTs; most studies small and short-term	Low (small RCTs, short duration)	Limited adult data; most studies small	Adjunctive only (infants/children)	(Ojetti et al., 2014; Coccorullo et al., 2010; Jadrešin et al., 2020)
Probiotics (single strain, Probio87)	*L. plantarum* Probio87, 12 weeks	ΔCSBM +1.21 vs. +0.42 in placebo at week 12; 100% achieved CSBM ≥3/week vs. 64% placebo	↑Bile acid levels 38% (*r* = 0.85)	Conference abstract (IDDF2025, *n* = 101)	Very low (preliminary, not peer-reviewed)	Conference abstract only; full data unavailable; awaiting peer-reviewed publication	Not ready (awaiting peer review)	[Zheng F. et al., 2025 (conference abstract)]
Probiotics (multi-strain)	*B. longum* + *L. bulgaricus* + *S. thermophilus* (triple-viable); *L. acidophilus* + *L. rhamnosus* + *L. reuteri* + *L. plantarum* + *B. animalis* (five-strain)	↓GITT in STC (*n* = 20, *p* = 0.012); improved intestinal transit rate and stool consistency in mice (n.s. effect size not reported)	Improved CSS scores; colonization persisted 2 weeks post-treatment for *L. plantarum* and *L. rhamnosus*	Small RCT (*n* = 20) + animal studies	Low (small human RCT, *n* = 20, plus animal data)	Small human trial (*n* = 20), no placebo control in one arm; animal data not directly translatable	Exploratory (needs larger RCTs)	(Luo et al., 2025; He et al., 2022)
Prebiotics (NextDext™)	Dextran prebiotic, 5 g t.i.d., 2 weeks	Bristol score 2.33 → 3.50 (*p* = 0.064, trend)	Restored opioid-associated dysbiosis (↑*B. longum*, *R. hominis*)	Small RCT (*n* = 24, 90% opioid users)	Low (very small trial, *n* = 12 per group, short duration)	Very small sample size, short duration; trend only, not statistically significant	Exploratory (opioid-associated constipation)	(Culqui Lévano et al., 2025)
Synbiotics	Probiotics + prebiotics (various); GT-optimized pairing	WMD + 1.31 (95% CI 0.62–2.00) for synbiotics vs. +0.93 for probiotics alone (Zhan et al., 2021); mouse: ↓first black stool time, ↑fecal water content	Restored colonic neurotransmitter homeostasis, enriched butyrate-producing bacteria	Meta-analysis + small RCTs + animal study	Low to moderate (meta-analysis suggests synbiotics > probiotics alone, but high heterogeneity)	Few dedicated RCTs; heterogeneous formulations; animal data require human validation	Exploratory	(Ding et al., 2024; Wang et al., 2026; Ding et al., 2016)
Postbiotics	ABB C24 (NextDext™ + heat-inactivated yeast); heat-inactivated *B. bifidum* MIMBb75	Bristol score 2.33 → 2.75; no direct bowel frequency effect size reported	↑Microbial diversity; ↓*M. smithii*; in IBS, global symptom improvement	Very small RCT (*n* = 24) + IBS RCT (not pure constipation)	Low to moderate (IBS RCT moderate; pure constipation evidence low)	Very small sample size for pure constipation evidence; IBS data not directly applicable	Exploratory (patients who cannot tolerate live probiotics)	(Culqui Lévano et al., 2025; Andresen et al., 2020)

WMD, weighted mean difference; CI, confidence interval; PAC-SYM, patient assessment of constipation symptoms; PAC-QOL, patient assessment of constipation quality of life; CFU, colony-forming unit; RCT, randomized controlled trial; FC, functional constipation; CSBM, complete spontaneous bowel movement; GITT, gastrointestinal transit time; STC, slow-transit constipation; CSS, constipation symptom score; GI, gastrointestinal; t.i.d., three times daily; GT, generation time; IBS, irritable bowel syndrome; IBS-C, constipation-predominant irritable bowel syndrome; FMT, fecal microbiota transplantation; GIQLI, Gastrointestinal Quality of Life Index; BSFS, Bristol Stool Form Scale; SBM, spontaneous bowel movement; GRADE, Grading of Recommendations Assessment, Development and Evaluation.

### 4.7 Comparative perspective on microbiota-based interventions

Table 4 below summarizes the comparative advantages, limitations, evidence strength and potential population applicability of the five intervention strategies discussed above. In brief: (1) Probiotics (particularly multi-strain formulations containing *Bifidobacterium* and *Lactobacillus*) have the largest body of evidence, but the overall quality remains low to very low with high heterogeneity. They may be considered as adjunctive therapy for mild-to-moderate FC. (2) Prebiotics (inulin 12 g/day) have moderate-quality evidence from a single well-designed trial, with baseline microbiota characteristics predicting response. (3) Synbiotics may offer synergistic effects, but evidence is limited and heterogeneous. (4) Postbiotics are exploratory but offer safety advantages for patients who cannot tolerate live probiotics. (5) FMT has shown promising results in meta-analyses, but remains investigational and should be reserved for refractory cases within research settings.

Collectively, microbiota-targeted interventions exhibit distinct strengths and limitations. While probiotics currently possess the most mature evidence base, the overall certainty of evidence remains low to moderate, and none can yet be routinely recommended as guideline-level therapy for constipation. The choice of intervention should be guided by patient phenotype, evidence quality, safety profile, and availability of resources.

## 5 Discussion

### 5.1 Critical appraisal of current evidence: heterogeneity, causality and evidence gaps

Before discussing future directions, we critically appraise the major limitations and sources of heterogeneity in the current literature. Overall, despite two decades of intensive research, the field suffers from low-to-moderate evidence certainty across most domains: microbiota signatures are inconsistent, MR provides genetic but not biological causation, mechanistic evidence is largely from animal models, and most intervention studies are small, short-term, and heterogeneous. This overarching limitation should be kept in mind when interpreting all subsequent discussions.

#### 5.1.1 Heterogeneity in microbiota studies

The inconsistency in alpha-diversity findings (some studies reporting reduced diversity in constipation, others not) can be attributed to several factors: (i) DNA extraction methods—methods without bead-beating (wall-breaking) underestimate Gram-positive bacteria such as *Bifidobacterium* and Firmicutes, affecting diversity estimates (Lim et al., 2018; Erhardt et al., 2023); (ii) constipation subtype—STC, FC and IBS-C may have different microbial profiles (Han et al., 2024; Roy et al., 2025; Chassard et al., 2012); (iii) sample type—mucosal microbiota differs from fecal microbiota (Parthasarathy et al., 2016; Quigley and Spiller, 2016); (iv) population characteristics—age, diet, geography, medication use (especially opioids and proton-pump inhibitors); and (v) sequencing methods—16S rRNA vs. metagenomics, variable region selection, sequencing depth. Future studies should standardize methodologies and report detailed metadata. It should also be noted that alpha-diversity alone is unlikely to serve as a robust biomarker for constipation and should be interpreted in conjunction with functional and metabolomic analyses.

#### 5.1.2 Reproducibility crisis

Despite numerous cross-sectional studies reporting microbiota alterations in constipation, a universally reproducible “constipation microbiome signature” has not emerged. This lack of reproducibility is not unique to constipation—it reflects broader challenges in microbiome research—but several specific factors contribute to the inconsistency in this field.

First, dietary variation is a major confounder. Diet is the primary determinant of gut microbiota composition, yet most constipation studies do not adequately control for or report habitual dietary fiber intake, fluid consumption, or other dietary factors (Zmora et al., 2019). Second, geographic and population differences contribute substantially: microbiota composition varies across ethnic groups and geographical regions, but most constipation studies are small and conducted in single centers, limiting generalizability (He et al., 2018). Third, medications—particularly proton-pump inhibitors (PPIs), metformin, non-steroidal anti-inflammatory drugs (NSAIDs), and opioids—strongly shape microbiota structure, but many studies do not systematically account for medication use, leading to unmeasured confounding (Vich Vila et al., 2020). Fourth, technical factors in microbiota profiling, including DNA extraction method (bead-beating vs. no bead-beating, which biases detection of Gram-positive bacteria), 16S rRNA variable region selection, sequencing depth, and bioinformatic pipelines, introduce substantial batch effects that obscure true biological signals. Even within the same study population, stool water content and colonic transit time may influence the recovered microbiota profile, as drier feces can alter the relative abundance of certain taxa independently of constipation status (Costea et al., 2017). Fifth, inter-individual variability in gut microbiota is inherently high; the human gut microbiome varies more across individuals than across many disease conditions, and constipation is a multifactorial, heterogeneous syndrome rather than a single disease entity. Consequently, constipation-associated microbial signals, if they exist, may be small in magnitude relative to background inter-individual variation (Integrative HMP (iHMP) Research Network Consortium, 2019).

Therefore, a universal “constipation microbiome signature” may not exist. Rather, constipation may be associated with context-dependent shifts in specific microbial modules or functional pathways that are not detectable at the taxonomic level across diverse populations. Future studies should prioritize harmonized methodologies, careful control of confounders, and sufficiently large, multi-center cohorts to improve reproducibility and identify robust, clinically actionable microbiome features.

#### 5.1.3 The methanogen paradox

Methane-producing archaea, particularly *M. smithii*, have frequently been associated with constipation and delayed intestinal transit. Experimental evidence suggests that methane may inhibit intestinal motility and prolong transit time (Hoegenauer et al., 2022; Culqui Lévano et al., 2025). However, the relationship remains controversial. Some studies, including a MR analysis by Zheng J. et al. (2025), have even reported a negative correlation between methanogen abundance and constipation risk (higher abundance associated with lower risk), and methane production itself may partly reflect prolonged intestinal transit rather than act as an initiating pathogenic factor. Thus, the methanogen-constipation relationship is likely bidirectional. Slower transit may create an anaerobic environment favorable for methanogen expansion, while methane production may further exacerbate dysmotility. In addition, there are several possible explanations: (i) host genetics—MR detects genetic proxies that may not reflect actual microbial abundance in all individuals; (ii) functional redundancy—not all methanogens produce equal amounts of methane; (iii) interaction with other microbes—methanogens often co-occur with specific hydrogen-producing bacteria; (iv) regional differences—colonic vs. fecal methanogen abundance may differ. This paradox highlights that cross-sectional associations and even MR findings must be interpreted with caution and validated experimentally.

#### 5.1.4 Limitations of MR studies (also relevant to Section 2.5)

While MR provides stronger causal inference than observational studies, it has several limitations: (i) genetic instruments explain limited variance—the IVs used for microbial traits often explain only a small fraction of abundance variation; (ii) horizontal pleiotropy—genetic variants may affect constipation through pathways independent of the microbiota; (iii) reverse causality still possible—MR cannot completely exclude bidirectional effects when IVs are weak; (iv) lack of species-level resolution—many MR studies use genus-level data; (v) generalizability—MR findings from European-ancestry cohorts may not apply to other populations (De Lillo et al., 2021). Therefore, MR should be viewed as providing “genetic-level causal inference” rather than proof of biological causation.

#### 5.1.5 Evidence strength across intervention studies

Table 3 presents a risk of bias assessment for key studies using an adapted Cochrane ROB-2/ROBINS-I framework, including a column on implications for interpretation. Overall, the evidence base for microbiota-based interventions in constipation is low to moderate, with few large, multi-center, double-blind, placebo-controlled RCTs of long duration. Many studies have small sample sizes, short follow-up, and high or unclear risk of bias in several domains. Consequently, the clinical recommendations that can be drawn are preliminary (see Section 5.6).

**Table 3:** Critical appraisal and risk-of-bias considerations in microbiota-targeted intervention studies.

Study (first author, year)	Study design	Domain 1: randomization/confounding	Domain 2: deviations from intended intervention	Domain 3: missing outcome data	Domain 4: outcome measurement	Domain 5: selective reporting	Overall risk of bias	Specific concerns/risk factors	Implications for interpretation
Ding et al., 2024	Meta-analysis of RCTs	Low (included RCTs had variable quality)	Low	Low	Low	Low	Moderate	High heterogeneity across included RCTs (*I*^2^ = 81–85%); publication bias cannot be fully excluded; GRADE evidence downgraded to low/very low	Results should be interpreted cautiously because heterogeneity limits generalizability; meta-analytic estimates may not apply to individual patients
Zhang C. et al. (2020)	Meta-analysis of RCTs	Low	Low	Low	Low	Low	Moderate	Moderate heterogeneity; relatively small number of RCTs per subgroup	Findings are supportive but not conclusive; larger, more homogeneous studies needed
Takeda et al., 2023	RCT (parallel)	Low (randomized, double-blind)	Low	Low (ITT analysis)	Low (validated scales)	Low	Low	Negative primary endpoint; subgroup analyses not pre-specified; potential over-interpretation of secondary outcomes	Primary analysis failed to show benefit; subgroup findings are hypothesis-generating, not confirmatory
Puhlmann et al., 2025	RCT (crossover)	Low (randomized, double-blind)	Low	Low	Low	Low	Low	Crossover design may introduce period-related carryover effects; baseline microbiota stratification not pre-specified	Carryover effect suggests potential period-related bias; results require replication in parallel-design RCT
He et al., 2022	Animal + human component	High (human part not randomized)	High	Low	Moderate (animal outcomes)	Unclear	High	Human component not randomized; animal data cannot be directly translated to human efficacy; unclear selective reporting	Human conclusions are not reliable; animal findings require human validation before clinical application
Culqui Lévano et al., 2025	RCT (parallel, small)	Unclear (randomization method not detailed)	Low	Low (small sample, completers only)	Low	Low	Some concerns	Very small sample size (*n* = 12 per group); short intervention duration (2 weeks); no ITT analysis; unclear randomization sequence generation	Results are very preliminary; small sample size and short duration limit generalizability; trend not statistically significant
Luo et al., 2025	Uncontrolled human trial (*n* = 20) + animal	High (no control group)	High	Low	Moderate	Unclear	High	No placebo control; small human sample size (*n* = 20); unclear outcome reporting; animal-human translation uncertain	Findings are exploratory; no causal conclusions can be drawn from the human component
Wang et al., 2025	Meta-analysis of FMT studies (*n* = 9)	Moderate (included studies heterogeneous)	Low	Low	Low	Low	Moderate	High heterogeneity across 9 included studies (*I*^2^ values vary); small sample sizes in primary studies; variable FMT protocols and follow-up durations	Evidence supports potential benefit, but high heterogeneity means effect estimates may not generalize; standardized protocols needed
Tan S. et al. (2025)	Network meta-analysis (*n* = 29 RCTs)	Moderate (primary studies low quality)	Low	Low	Low	Low	Moderate	Low-quality primary studies; network meta-analysis relies on indirect comparisons; potential inconsistency between direct and indirect evidence	Network meta-analysis findings are only as reliable as the primary studies; indirect comparisons may introduce bias
Zheng F. et al. (2025)	RCT (conference abstract only, *n* = 101)	Unclear (insufficient reporting)	Unclear	Unclear	Unclear	Unclear	High	Conference abstract only; no full peer-reviewed publication; insufficient methodological details (randomization, blinding, allocation concealment, ITT not reported)	Cannot be used for clinical guidance; interpret as preliminary and hypothesis-generating only
He et al. (2024)	MR study (two-sample)	Low (genetic IVs)	Low (not applicable)	Low	Low	Low	Low for genetic inference	Does not prove biological causation; IVs explain limited variance in microbial exposure; potential horizontal pleiotropy cannot be fully excluded	Supports genetic-level causal inference but does not establish biological causation; requires experimental validation
Zhou et al. (2024)	MR study (bidirectional)	Low (genetic IVs)	Low	Low	Low	Low	Low for genetic inference	Reverse causality cannot be fully excluded with weak IVs; lack of species-level resolution (genus-level data only)	As above; directional inference is genetic, not biological

RCT, randomized controlled trial; ITT, intention-to-treat analysis; IVs, instrumental variables; MR, Mendelian randomization; FMT, fecal microbiota transplantation; *I*^2^, measure of statistical heterogeneity; GRADE, Grading of Recommendations Assessment, Development and Evaluation. This table adapts the Cochrane ROB-2 tool for randomized trials and ROBINS-I for non-randomized studies. For meta-analyses, risk of bias reflects the quality and heterogeneity of included primary studies. For MR studies, “low for genetic inference” indicates validity of the genetic instrument, not biological causation. “High” risk of bias in conference abstracts reflects insufficient reporting rather than necessarily flawed conduct; such studies should be interpreted as preliminary and hypothesis-generating.

**Table 4:** Comparative overview of microbiota-based interventions for constipation.

Intervention	Evidence strength (highest available)	Recommendation grade (clinical)	Key advantages	Key limitations	Suitable population (exploratory)	Reference dosage	Suggested follow-up duration	References
Probiotics	Low to very low (GRADE)	Conditional (adjunctive)	Largest body of evidence; multi-strain may be more effective	High heterogeneity (*I*^2^ = 81–85%); strain-specific effects; short-term studies	Mild-to-moderate FC; adjunctive only	10^9^–10^11^ CFU/day (strain-dependent)	≥4 weeks	(Zhang C. et al., 2020)
Prebiotics (inulin)	Moderate (single RCT)	Conditional (adjunctive)	Good safety profile; carry-over effect; baseline microbiota predicts response	Single study only; carry-over effect may indicate period-related bias	FC with high baseline butyrate producers	12 g/day	≥4 weeks	(de Preter et al., 2008)
Synbiotics	Low to moderate	Exploratory	Potential synergistic effect	Few dedicated RCTs; heterogeneous formulations; animal data require human validation	Exploratory	Variable; GT-optimized pairing proposed	≥4–8 weeks	(Zhang C. et al., 2020; Ding et al., 2016; Kim et al., 2021)
Postbiotics	Low	Exploratory	Safety advantage (no live bacteria); increased diversity	Very small sample size; IBS data not directly applicable	Patients who cannot tolerate live probiotics	Variable (ABB C24: 5 g t.i.d.)	≥2–4 weeks	(Culqui Lévano et al., 2025; Marasco et al., 2024)
FMT	Low to moderate	Investigational (research setting only)	Ranked #1 in network meta-analysis for SBM, CSBM, BSFS	High heterogeneity; small sample sizes; variable protocols; long-term safety data lacking; investigational	Refractory constipation (research setting only)	Variable (standardized protocols not yet established)	≥3–6 months; long-term safety ≥2 years needed	(Tan S. et al., 2025; Li et al., 2022; Tan W. et al., 2025)

The populations listed are exploratory suggestions based on available evidence; actual selection should consider individual patient phenotype, comorbidities, and preferences. References (Tan et al., 2025) are conference abstracts and should be interpreted as preliminary data. Recommendation grades reflect the authors’ interpretation of current evidence and are intended to guide clinical decision-making: “Conditional/adjunctive” indicates that the intervention may be considered as an adjunctive therapy in selected patients; “Exploratory” indicates that evidence is insufficient to recommend routine use; “Investigational” indicates that the intervention should be restricted to research settings. Follow-up durations are suggested minimums for assessing efficacy; longer durations are needed to evaluate durability and safety.

#### 5.1.6 Research landscape summary

**Figure 2:**
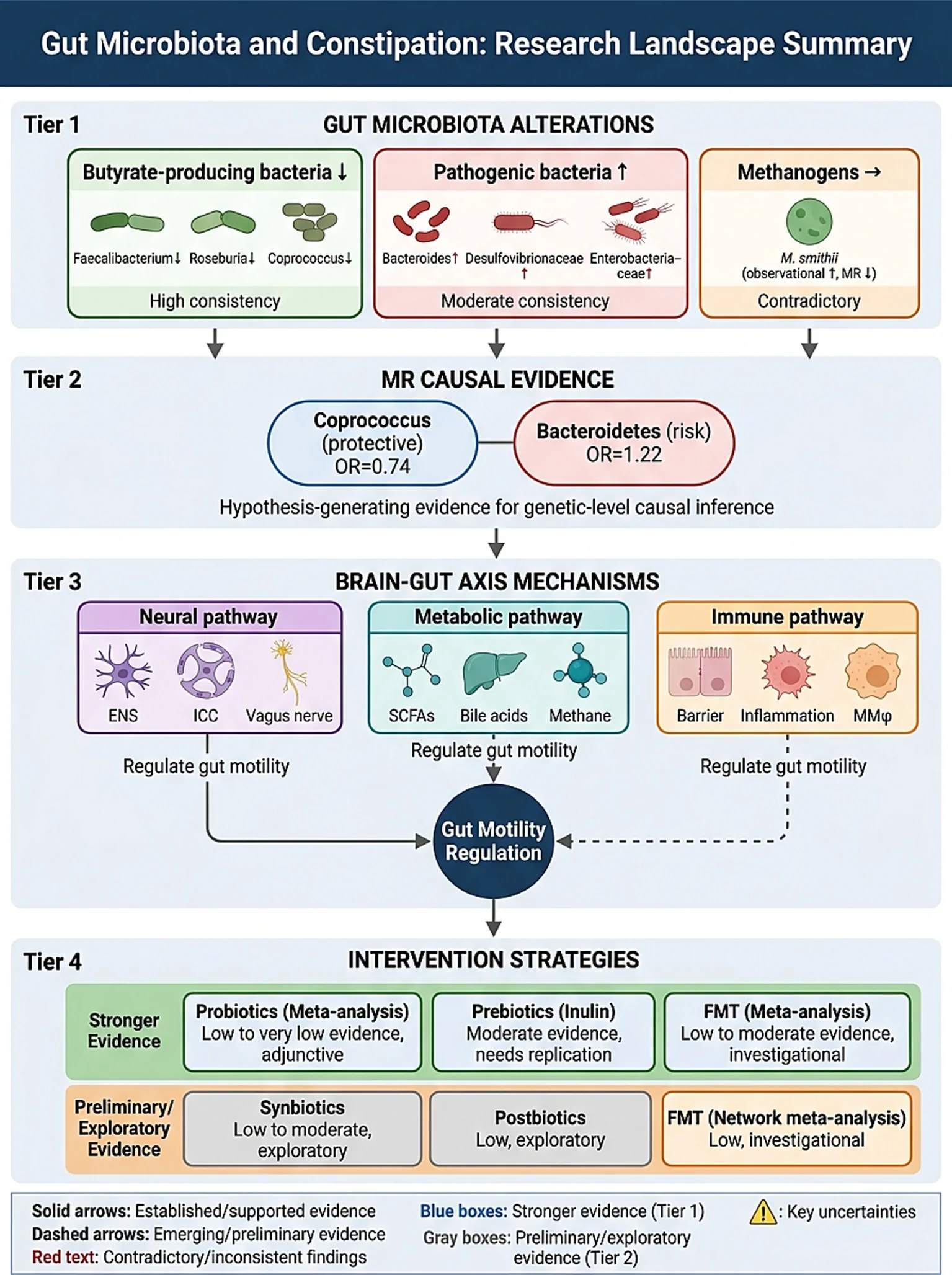
Gut microbiota and constipation: research landscape summary.

Figure 2 provides a graphical summary of the current research landscape on gut microbiota and constipation. The figure illustrates the integrated framework from microbiota alterations (butyrate-producing bacteria↓, pathogenic bacteria↑, methanogens → complex association) through MR evidence (causal inference for *Coprococcus* protective, Bacteroidetes risk) and BGA mechanisms (neural, metabolic, immune pathways) to intervention strategies (probiotics, prebiotics, synbiotics, postbiotics, FMT), with evidence tiering (stronger vs. preliminary) and key uncertainties indicated. This visual summary is intended to help readers quickly grasp the overarching structure and evidence distribution of the field.

### 5.2 Sources of research heterogeneity and coping strategies

Differentiation of constipation subtypes is a primary step to reduce heterogeneity. Future studies should clearly report diagnostic criteria and stratify by subtype.

Standardization of microbiota detection methods is essential. DNA extraction methods (especially bead-beating) significantly affect Gram-positive bacterial detection (Lim et al., 2018; Erhardt et al., 2023). Primer choice, variable region, sequencing depth and bioinformatic pipelines also influence results. Metagenomics provides species-level and functional information but is costly (Han et al., 2024; Tian et al., 2021). Use of standardized operating procedures and detailed methodological reporting is recommended.

### 5.3 Exploration of individualized therapy

Baseline microbiota characteristics may predict intervention response, opening possibilities for personalized therapy.

Beyond microbiota profiling, emerging evidence suggests that host genotype, dietary patterns, and concomitant medication use may influence responses to microbiota-based interventions. Host genetic variants in SCFA receptors (e.g., GPR41, GPR43) have been shown to modulate the biological effects of SCFAs on gut motility and may contribute to inter-individual variability in treatment response. Dietary factors such as fiber intake type and amount are well-established determinants of SCFA production and can substantially affect baseline microbiota composition (Zmora et al., 2019). Regarding medication use, PPIs have been associated with reduced gut microbial diversity and alterations in specific taxa, including decreased *Bifidobacterium* and increased *Enterococcus*, which could potentially modify the efficacy of probiotic interventions (Vich Vila et al., 2020). Similarly, metformin has been shown to enhance *Bifidobacterium* abundance and increase SCFA production, suggesting that patients taking metformin may have a different baseline microbiota profile that could influence intervention outcomes (Vich Vila et al., 2020). Although direct evidence linking these factors to constipation-specific intervention responses remains limited, these observations highlight the need for careful phenotyping and consideration of host and environmental factors in future studies.

#### 5.3.1 Prebiotic response prediction

Puhlmann et al. (2025) showed that inulin responders had higher baseline butyrate producers (*Faecalibacterium*, *Roseburia*) and lower *Bifidobacterium*. This aligns with de Preter et al. (2008) that baseline *Bifidobacterium* activity influences prebiotic response and Bouhnik et al. (2004) that baseline counts influence probiotic-stimulated *Bifidobacterium* proliferation.

#### 5.3.2 Probiotic response prediction

Gargari et al. (2024) found that *C. aerofaciens* may predict response to probiotic therapy in non-constipated IBS. Martoni et al. (2019) found that gender and Bristol score were associated with beta diversity. Future studies could use baseline microbiota to screen patients likely to benefit.

#### 5.3.3 Enterotype-guided interventions

Vandeputte et al. (2016) revealed that stool consistency is closely linked to enterotype and bacterial growth rates. Vervier et al. (2022) identified two IBS microbiota subtypes with different responses to a low FODMAP diet. These findings provide a basis for enterotype-based stratification.

Beyond host genotype and environmental factors, constipation subtype itself may influence intervention response. For example, STC patients with reduced butyrate producers may preferentially benefit from prebiotics that enhance SCFA production, whereas IBS-C patients with elevated Enterobacteriaceae may respond better to probiotics that compete with pathogenic taxa. However, direct evidence for subtype-guided intervention selection is currently lacking, and this represents a priority area for future phenotype-stratified RCTs.

### 5.4 Toward a microbiota-driven mechanistic framework for constipation

Based on the evidence discussed, we propose an integrated conceptual framework: the microbiota-brain-gut dysregulation loop. Rather than a simple linear cause-effect, constipation may arise from a self-reinforcing cycle involving three interconnected loops:

#### 5.4.1 Neural loop

Dysbiosis reduces butyrate-producing bacteria → decreased butyrate → impaired ICC network (reduced c-kit/SCF signaling) → diminished slow wave activity → reduced ENS neuronal excitability → slower colonic transit → prolonged stool retention → further dysbiosis.

#### 5.4.2 Metabolic loop

Reduced SCFAs and secondary BAs → decreased TGR5-mediated 5-HT release from EC cells → impaired peristaltic reflex → slowed transit → reduced fiber fermentation → further SCFA reduction. Concurrently, altered bile acid metabolism may reduce TGR5/FXR signaling, exacerbating the problem.

#### 5.4.3 Immune loop

Dysbiosis increases gut permeability → low-grade systemic inflammation → activation of MMφ → production of inflammatory cytokines that inhibit ENS and ICC function → further motility reduction.

These loops are not independent; they interact at multiple points. For example, butyrate not only directly enhances ICC activity but also strengthens barrier function, reducing immune activation. 5-HT released via SCFAs and BAs also modulates ENS development and inflammation. Prolonged intestinal transit may favor methane-producing organisms, whose metabolites may further suppress intestinal motility. Dysbiosis-associated barrier dysfunction and low-grade inflammation may impair enteric neurons and ICC networks, further amplifying dysmotility.

Together, these processes may establish a bidirectional microbiota-motility dysfunction loop that contributes to chronic constipation. This framework may also help explain why microbiome signatures differ across constipation phenotypes and why single-target interventions often produce inconsistent therapeutic responses.

This framework suggests that effective therapy may need to target multiple loops simultaneously. It also predicts that baseline microbiota characteristics (e.g., butyrate-producer abundance, bile acid-transforming capacity) will determine which loop is dominant in a given patient, guiding personalized intervention (e.g., SCFA-enhancing prebiotics vs. bile acid-modulating probiotics vs. barrier-repairing postbiotics). Future experimental studies should test these hypotheses using gnotobiotic models and multi-omics approaches.

### 5.5 Future research directions

#### 5.5.1 Mechanism level

Move from correlation to causal verification using sterile animal colonization experiments and metabolite supplementation (Obata and Pachnis, 2016). Integrate metagenomics with metabolomics to reveal microbiota-metabolite-phenotype networks (Zheng F. et al., 2025; Wang et al., 2026). Spatial metabolomics can localize metabolites to specific tissue regions, offering new perspectives on brain-gut communication (Qian et al., 2023).

#### 5.5.2 Clinical level

Large, multi-center, long-term RCTs using uniform Rome IV criteria and FDA/EMA-recommended endpoints are needed. Detailed reporting of strain information, dose, regimen and baseline microbiota is essential. Long-term safety data (especially for FMT) are lacking.

#### 5.5.3 Technical level

Machine learning can improve predictive models (Chen et al., 2021; Marcos-Zambrano et al., 2021). Capsule endoscopy offers the possibility of sampling microbiota from different intestinal segments (Wensel et al., 2022). CRISPR-Cas technology could be used to engineer probiotics with enhanced functions (Roberts and Barrangou, 2020).

#### 5.5.4 Intervention level

Personalized interventions based on microbiota typing are the way forward. Postbiotics deserve attention for their safety advantages (Culqui Lévano et al., 2025). Phage therapy may enable targeted removal of specific pathogenic bacteria (Paule et al., 2018). Dietary interventions (e.g., low FODMAP diet) should be individualized based on microbiota characteristics (Vervier et al., 2022).

### 5.6 Potential clinical implications and future translational perspectives

Current evidence does not yet support guideline-level microbiota-based treatment recommendations for constipation. Nevertheless, selected microbiota-targeted interventions may offer potential adjunctive benefit in carefully selected patients.

Multi-strain probiotic formulations containing *Bifidobacterium* and *Lactobacillus* species have shown modest improvements in some clinical studies, although strain specificity and inter-individual variability remain important limitations.

Prebiotics such as inulin may improve bowel frequency and stool consistency in some individuals, particularly when baseline microbiota composition favors SCFA production.

FMT remains investigational and should currently be restricted to research settings or highly specialized centers.

Future precision-medicine approaches integrating microbiome profiling, metabolomics, and constipation phenotyping may improve individualized therapeutic strategies.

## 6 Conclusion

In this narrative review, we critically appraised the relationship between gut microbiota and constipation. The main conclusions are as follows.

Characteristic microbiota alterations exist in patients with constipation: decreased butyrate-producing bacteria (*Faecalibacterium*, *Roseburia*, *Coprococcus*), increased potentially pathogenic bacteria (*Bacteroides*, *Desulfovibrionaceae*), and complex associations with methanogens. Different constipation subtypes (STC, FC, IBS-C) show different profiles, and mucosal and fecal microbiota differ.

MR studies provide genetic-level causal inference suggesting that some microbiota alterations (e.g., *Coprococcus* as protective, Bacteroidetes as risk) may precede constipation. However, it must be re-emphasized that MR does not prove biological causation; these findings are hypothesis-generating and require experimental validation.

The BGA is a key mechanistic link. Neural (ENS, ICC, vagus), metabolic (SCFAs, BAs, methane) and immune (barrier, low-grade inflammation, MMφ) pathways mediate microbiota-host interactions. 5-HT is a central signaling molecule, with SCFAs promoting 5-HT synthesis and BAs regulating 5-HT release via TGR5.

Probiotics have shown efficacy in improving constipation in some RCTs and meta-analyses, but the evidence quality is low to very low, with high heterogeneity. Efficacy is strain-specific, and multi-strain formulations may be more effective. Colonization capacity varies among strains.

Prebiotics, synbiotics, postbiotics and FMT each show promise but remain largely investigational. Inulin (12 g/day) improved bowel frequency and quality of life in one well-designed trial, with baseline microbiota predicting response. Postbiotics (e.g., ABB C24) reduced *M. smithii* and increased diversity. FMT has shown promising results in meta-analyses; however, since most current studies are small in scale, of short duration, and vary in methodology, it should still be considered a subject of further research regarding constipation.

Personalized treatment is a future direction. Baseline microbiota characteristics (e.g., butyrate producer abundance, *Bifidobacterium* counts) may predict intervention response. Enterotyping and machine-learning-assisted microbiota analysis may advance precision medicine.

Overall, while the gut microbiota is increasingly recognized as an important contributor to constipation pathophysiology, the evidence for causality remains incomplete, and most microbiota-based interventions require further validation in large, well-designed RCTs before routine clinical application. Standardization of methods and critical appraisal of heterogeneity are urgently needed. Future progress will likely depend on integrating microbiome profiling with metabolomics, host physiology, and phenotype-specific clinical stratification.
